# Causal Pathways from Blood Pressure to Larger QRS Amplitudes: a Mendelian Randomization Study

**DOI:** 10.1038/s41598-018-24002-0

**Published:** 2018-04-11

**Authors:** M. Yldau Van Der Ende, Tom Hendriks, Dirk J. Van Veldhuisen, Harold Snieder, Niek Verweij, Pim Van Der Harst

**Affiliations:** 1 University of Groningen, University Medical Center Groningen, The department of Cardiology, Groningen, The Netherlands; 2 University of Groningen, University Medical Center Groningen, The department of Epidemiology, Groningen, The Netherlands

## Abstract

Abnormal QRS duration and amplitudes on the electrocardiogram are indicative of cardiac pathology and are associated with adverse outcomes. The causal nature of these associations remains uncertain and could be due to QRS abnormalities being a symptom of cardiac damage rather than a factor on the causal pathway. By performing Mendelian randomization (MR) analyses using summary statistics of genome wide association study consortia with sample sizes between 20,687 and 339,224 individuals, we aimed to determine which cardiovascular risk factors causally lead to changes in QRS duration and amplitude (Sokolow-Lyon, Cornell and 12-leadsum products). Additionally, we aimed to determine whether QRS traits have a causal relationship with mortality and longevity. We performed inverse-variance weighted MR as main analyses and MR-Egger regression and weighted median estimation as sensitivity analyses. We found evidence for a causal relationship between higher blood pressure and larger QRS amplitudes (systolic blood pressure on Cornell: 55SNPs, causal effect estimate per 1 mmHg = 9.77 millimeters·milliseconds (SE = 1.38,*P* = 1.20 × 10^−12^) and diastolic blood pressure on Cornell: 57SNPs, causal effect estimate per 1 mmHg = 14.89 millimeters·milliseconds (SE = 1.82,*P* = 3.08 × 10^−16^), but not QRS duration. Genetically predicted QRS traits were not associated with longevity, suggesting a more prominent role of acquired factors in explaining the well-known link between QRS abnormalities and outcome.

## Introduction

**Figure 1:**
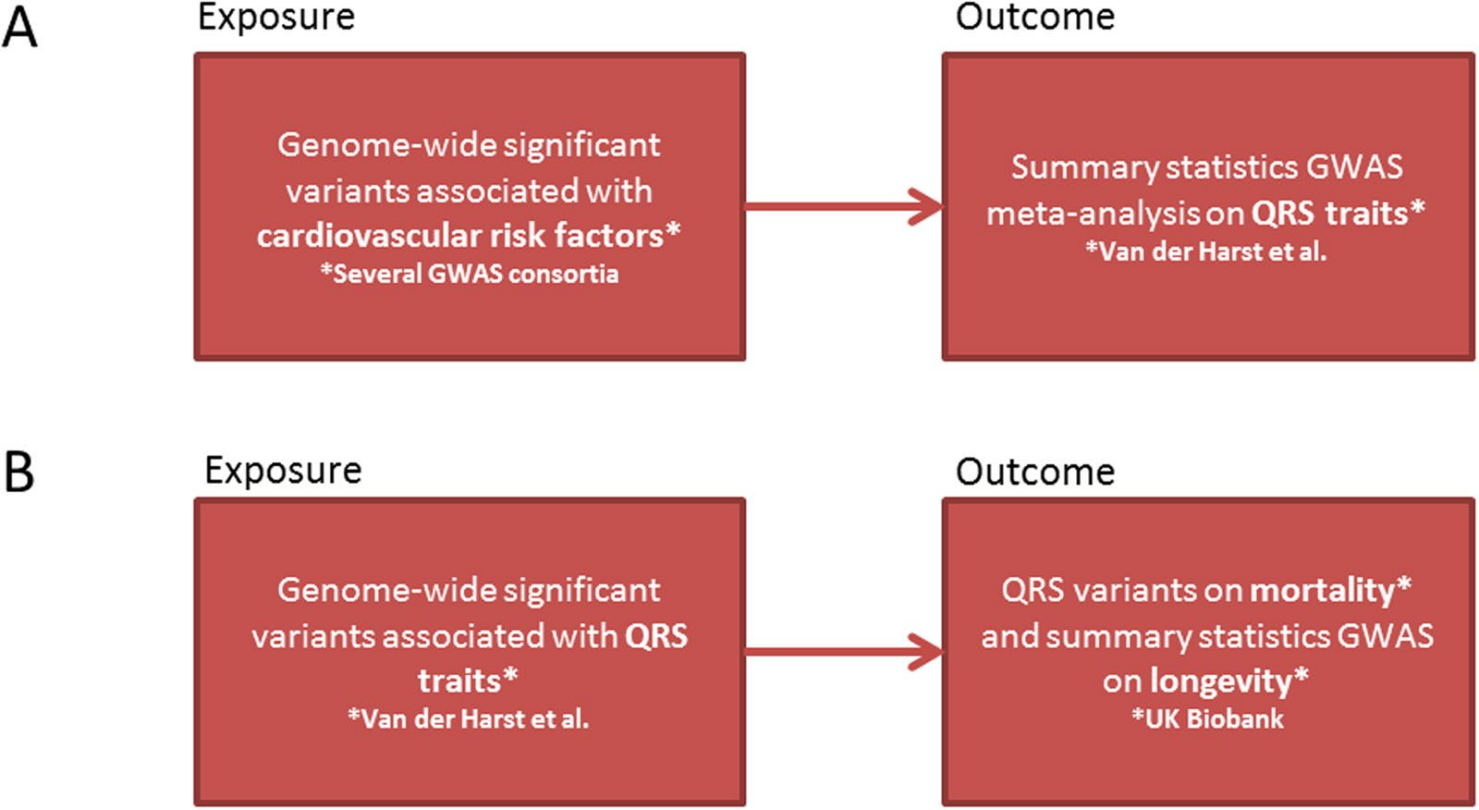
Flow charts of the MR analyses performed. (**A**) Is the schematic presentation of the MR analyses of cardiovascular risk factors on QRS traits to determine which cardiovascular risk factors causally lead to changes in QRS duration and QRS amplitude. (**B**) Is the schematic presentation of the MR analyses of QRS traits on mortality and longevity to determine whether a causal relationship of QRS traits with mortality and longevity exists.

A central feature of the electrocardiogram (ECG) is the QRS complex, reflecting ventricular depolarization. Abnormalities in the duration and amplitudes of the QRS complex are indicative of (early) cardiac pathology, including disorders of the conduction system and cardiac hypertrophy^1–3^. Abnormal QRS duration and amplitudes have also been associated with an increased risk of cardiovascular events and mortality^3–10^. The causal nature of these associations remains uncertain and changes in QRS duration and amplitudes could be a symptom of cardiac damage or a factor on the causal pathway. In this study, we aimed to determine which cardiovascular risk factors causally lead to QRS changes (Fig. 1A). Additionally, we aimed to determine whether a causal relationship of *genetically predicted* QRS traits with mortality and longevity exists (Fig. 1B). Mendelian randomization (MR) analyses are designed to investigate the causal nature of the relationship between risk factors and outcomes in observational data in the presence of confounding factors^11^. Using genetic variants as instruments, which are randomly assigned when passed from parents to offspring during meiosis, the genotype distribution in the population should be unrelated to the presence of confounders. To date, no such approach has been used to study the causal influence of risk factors on QRS abnormalities on the one hand and the influence of *genetically predicted* variation in QRS duration and amplitudes on mortality and longevity on the other hand. We recently conducted a large meta-analysis of genome wide association studies (GWAS) for QRS duration and amplitude yielding 52 loci for these traits (n = 73,518, explaining 2.7%, 3.2%, 4.1% and 5.0% of the variance of Sokolow-Lyon, Cornell, 12-lead sum and QRS duration, respectively)^12^. The summary statistics of this GWAS meta-analysis were used as outcome (cardiovascular risk factors on QRS traits) or exposure (QRS traits on mortality and longevity) in our MR analyses.

## Results

### Association between cardiovascular risk factors and QRS traits

**Figure 2:**
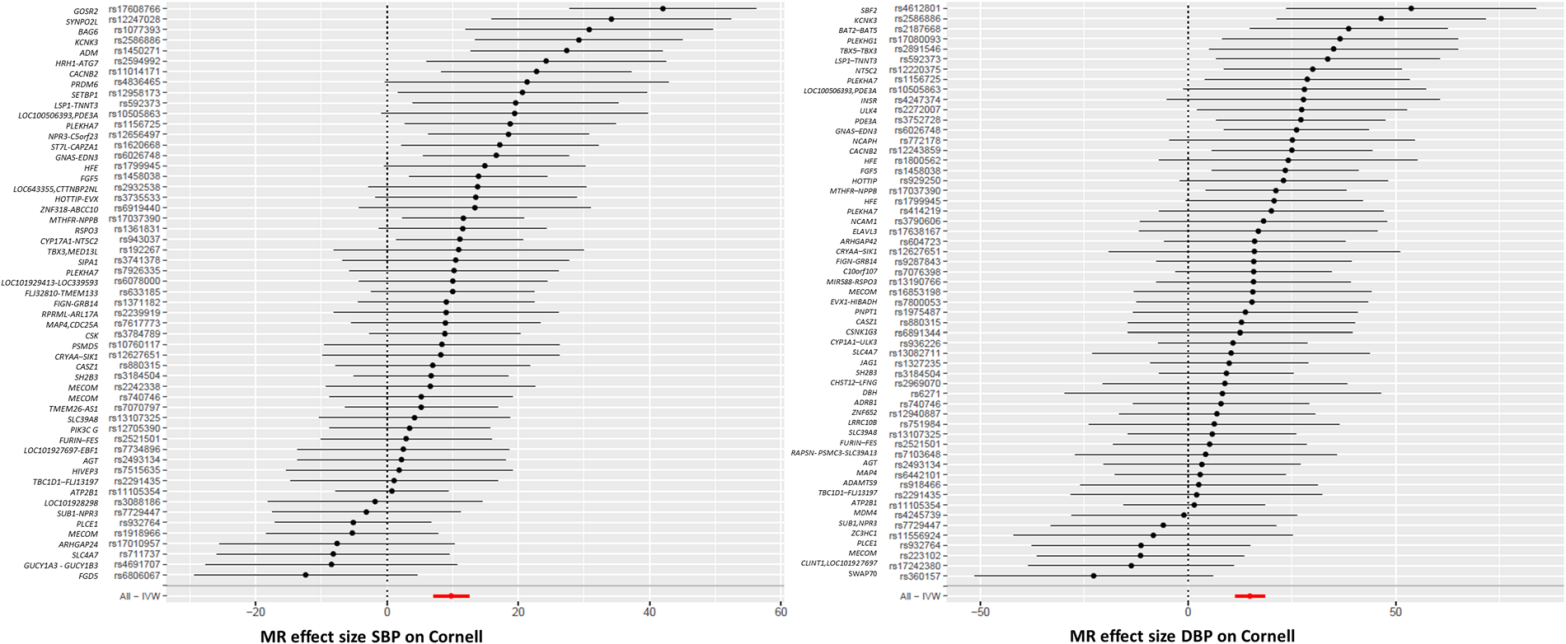
Forest plots: SBP and DBP associated with ECG Cornell criteria. On the X-axis the Mendelian Randomization effect size of blood pressure on Cornell product were displayed. On de Y-axis the different genetic variants were listed. DBP = diastolic blood pressure, MR = Mendelian randomization, SBP = systolic blood pressure.

**Figure 3:**
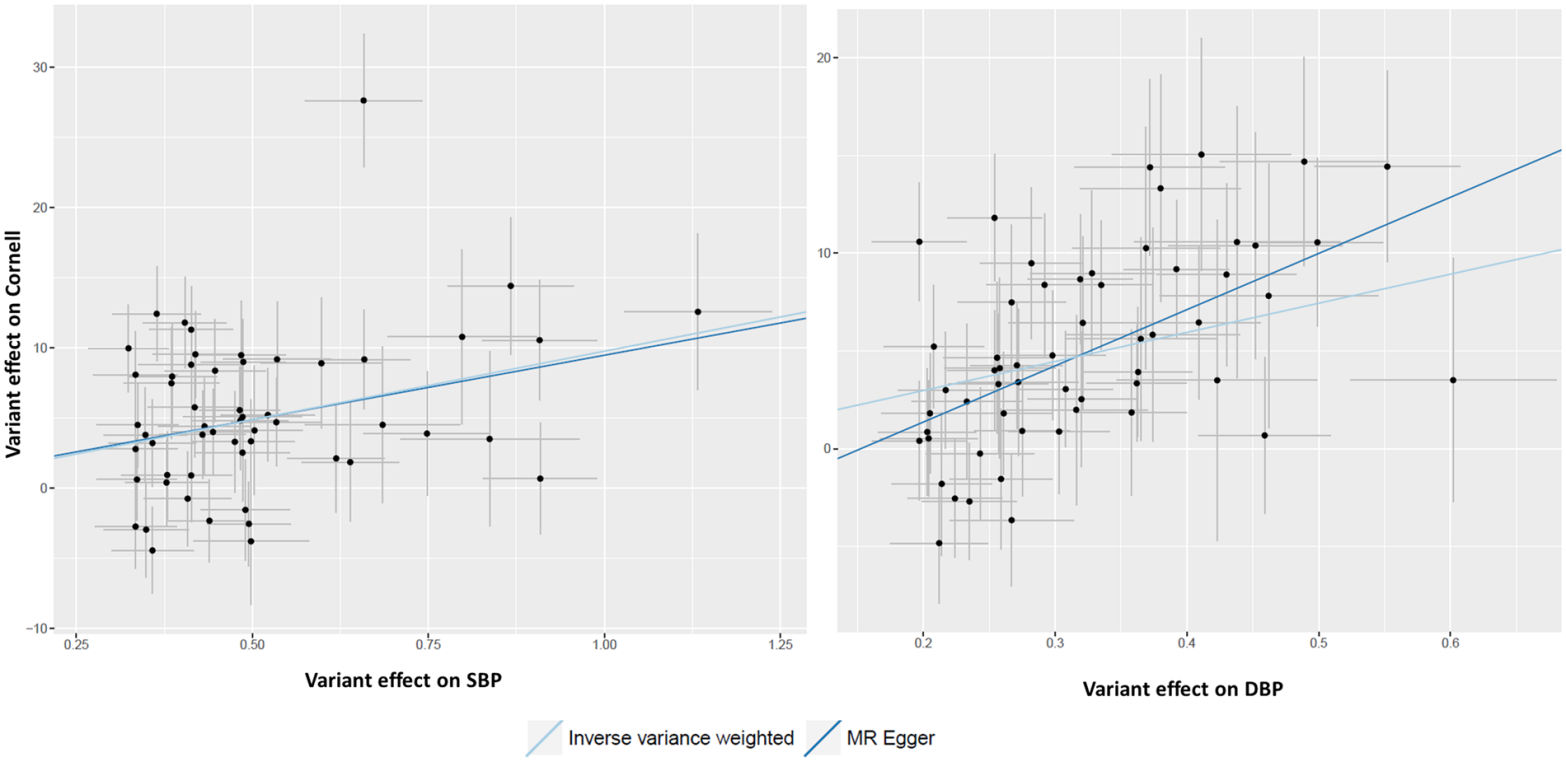
Scatter plots: SBP and DBP associated with ECG Cornell criteria. On the X-axis the variant effects on blood pressure are displayed and on the Y-axis the variant effect on Cornell product. The light blue line is the regression line of the inverse-variance-weighted fixed-effects meta analyses. The dark blue line is the regression line of the MR Egger regression line. DBP = diastolic blood pressure, MR = Mendelian randomization, SBP = systolic blood pressure.

Genetic variants for QRS duration and amplitude were extracted from the summary statistics of a large-scale GWAS and replication study of these phenotypes^12^. Table 1 and Supplementary Table 1 provide overviews of the combined effects of genetic variants previously associated with cardiovascular risk factors and their association with QRS duration and amplitude. Genetically predicted higher systolic blood pressure was associated with larger QRS amplitudes (Sokolow-Lyon (β 15.21, SE 2.38); Cornell (β = 9.77, SE = 1.38) and 12-lead sum (β = 86.35, SE = 12.45)) but not QRS duration. Also, genetically predicted higher diastolic blood pressure was associated with larger QRS amplitudes (Sokolow-Lyon (β = 23.20, SE = 3.76); Cornell (β = 14.89, = SE 1.82) and 12-lead sum (β = 111.28, SE = 18.81), but again not with QRS duration. The displayed β’s are in millimeters·milliseconds units. Figures 2 and 3 display the forest and scatter plots of systolic and diastolic blood pressure with ECG Cornell criteria, since these associations were most significant. The weighted median estimate confirmed the results. The MR Egger intercepts for analyses between blood pressure and QRS amplitudes were within the confidence interval of zero (P-values > 0.10), except from diastolic blood pressure on Cornell product. Searching for known pleiotropic effects of the blood pressure associated variants in the GWAS catalogue yielded little already known pleiotropic loci. In total, nine systolic or diastolic blood pressure loci had pleiotropic effects on other traits (loci in *FURIN-FES* associated with myocardial infarction, TMEM26-AS1 with Takotsubo, *SH2B3* with white blood cells, *SLE39A8* with body mass index and *ZNF318-ABCC10* and *ZC3HC1* with platelet count, *ULK4* with multiple myeloma, *BAT2-BAD5* with colitis ulcerosa and *MDM4* with breast cancer). Excluding these loci from our analyses did not change the significance. Because the heterogeneity P-values for the associations between blood pressure and amplitudes (except from diastolic blood pressure on Cornell product) were all smaller than 0.05 the analyses were repeated without the individually significant genetic variants (Supplementary files, Table 2 and Figs 1–5), after which heterogeneity P-values were all above 0.705. All of the associations between genetically predicted blood pressure and QRS amplitudes remained significant after exclusion of these individually significant genetic variants. The estimated statistical power for our MR analyses of blood pressure on QRS amplitudes were all above 0.80. No other associations between cardiovascular risk factors (measures of obesity, lipids and glucose levels, diabetes and smoking) and QRS traits were identified (P-values > 9.62 × 10^−5^, Supplementary Table 1).

**Table 1:** Mendelian Randomization analyses of cardiovascular risk factors and QRS traits.

Risk factor	QRS trait	P-value IVW	β (SE)	P-value Weighted median	β (SE)	MR Egger intercept	P-value	Heterogeneity P-value
SBP	QRS duration	1.60 × 10^−2^						
	Sokolow-Lyon	**1.73 × 10** ^**−10**^	15.21 (2.38)	**2.59 × 10** ^**−9**^	13.69 (2.28)	0.44 (4.05)	0.914	1.74 × 10^−13^
	Cornell	**1.20 × 10** ^**−12**^	9.77 (1.38)	**8.56 × 10** ^**−10**^	9.01 (1.47)	0.30 (2.34)	0.900	3.97 × 10^−5^
	12-lead sum	**3.99 × 10** ^**−12**^	86.35 (12.45)	**6.69 × 10** ^**−14**^	89.02 (11.88)	−5.15 (21.19)	0.809	1.95 × 10^−13^
DBP	QRS duration	4.33 × 10^−2^						
	Sokolow-Lyon	**6.96 × 10** ^**−10**^	23.20 (3.76)	**2.19 × 10** ^**−12**^	26.26 (3.74)	1.08 (4.28)	0.801	1.82 × 10^−11^
	Cornell	**3.08 × 10** ^**−16**^	14.89 (1.82)	**1.93 × 10** ^**−10**^	15.50 (2.43)	−4.38 (1.99)	**0.032**	1.07 × 10^−1^
	12-lead sum	**3.28 × 10** ^**−9**^	111.28 (18.81)	**1.78 × 10** ^**−10**^	121.89 (19.11)	−15.52 (21.31)	0.470	1.61 × 10^−9^

β = beta (in millimeters·milliseconds); DBP = Diastolic Blood Pressure; SBP = Systolic Blood Pressure; SE = Standard Error.

### Association between QRS traits and mortality and longevity

MR analyses were performed to determine the association of QRS duration and amplitudes with mortality and longevity. Genetic and follow-up data of 143,193 participants of the UK Biobank was available. During a median follow-up of 6.9 years (IQR 6.3–7.6 years), 4,372 participants died, of which 2,653 due to a cardiovascular cause. None of the genetically predicted QRS traits were associated with all-cause mortality or cardiovascular mortality (Supplementary file, Table 3). MR analyses between QRS traits and longevity also provided no evidence of association (Supplementary file, Table 3). We performed further sensitivity analyses by adding additional instrumental variables with higher P-values (up to P < 1 × 10^−4^) for QRS-traits to the MR analyses but this did not change the observations (*Supplementary file*, Table 3). The estimated statistical power for the MR analyses of QRS traits on mortality was below 0.80 for all QRS traits, but the estimated statistical power for the MR analyses of QRS traits on longevity was always above 0.80.

## Discussion

We found evidence for a causal relationship between higher systolic and diastolic blood pressure and larger QRS amplitudes (Sokolow-Lyon, Cornell and 12-lead sum products) but not QRS duration. No associations between other cardiovascular risk factors and QRS traits were found. Genetically predicted QRS traits were not associated with longevity. Our analyses of QRS traits on mortality were underpowered and could be repeated when more follow-up data of the UK Biobank will be available. In traditional population-based cohort studies, multiple associations between cardiovascular risk factors and larger QRS amplitudes and duration have been established, but whether these associations are causal is less clear. By studying the genotypes affecting blood pressure, we established evidence for a causal relationship of blood pressure with larger QRS amplitudes, but not with an increase in QRS duration. Larger QRS amplitudes are possibly more directly related to cardiac mass in response to increased blood pressure compared to QRS duration. Blood pressure and larger QRS amplitude and duration have been associated in multiple studies investigating the *phenotype* of blood pressure^13–16^. The difficulty is that the well-established relationship between higher blood pressure and QRS duration reported earlier may not be causal and might be a consequence of confounding factors both affecting blood pressure and QRS duration. For example, body mass index, both affecting blood pressure as well as QRS amplitudes^17^. In this study, we now found evidence for a causal relationship. In our MR analyses, no other cardiovascular risk factors (measures of obesity, lipids and glucose levels, diabetes and smoking) were associated with QRS duration or amplitudes, suggesting the absence of a direct causal relationship. Associations between the *phenotypes* of body mass index, diabetes and smoking with ventricular hypertrophy have well been described^18,19^. These observed links might be due to acquired or confounding risk factors or could very well be driven by the clustering of these risk factors with hypertension, since less than 20% of hypertension occurs without the presence of another cardiovascular risk factor^20^. Additionally, measured QRS amplitudes in individuals with a high body mass index will be lower due to increased chest wall and pericardial fat mass^17^, making it more difficult to detect signs of ventricular hypertrophy on ECG. One limitation of our MR approach is that the variants used were derived from a GWAS on QRS duration and amplitudes which was adjusted for body mass index^12^, possibly explaining the absence of associations between genetically predicted body mass index and QRS traits. In our study, genetically predicted QRS traits were not associated with longevity in the general population. *Phenotypes* of QRS duration and amplitude are well-known predictors of cardiovascular disease and mortality^21–25^, but a direct causal relationship has not been described and could be confounded by other factors. Additionally, no attempt has been made to differentiate between the effects of primary versus secondary QRS abnormalities. Our findings suggest that the reported association between QRS traits and mortality (in populations with medical conditions) may indeed have resulted more from the (confounding) environmental risk factors than the genetic component of QRS duration and amplitudes itself. Further, associations between QRS traits and mortality or longevity could very well be driven by extreme cases (such as QRS duration > 120 ms) that fall out of range of the genetically predicted variation.

Some general limitations of our MR analyses should be considered as these rely on three key assumptions. First, the genetic variants must associate with the risk factor of interest. Second, the genetic variants may not associate with potential confounders. Third, the genetic variants may only affect the outcome via the risk factor of interest^11^. To be a valid instrumental variable, a genetic variant should be associated only with the respective risk factor. The genetic variants used in our analyses were genome wide significant associated with the risk factors and by calculating F-statistics for the genetic variants we demonstrated that our analyses were unlikely to be biased due to weak instruments. For systolic and diastolic blood pressure, there is an overlap of the genetic variants (19 variants), which make it more difficult to discriminate the instrumental variants of systolic blood pressure and diastolic blood pressure correctly from each other. Excluding these genetic variants from our analyses would lead to a loss of strength of our MR analyses. By performing sensitivity analyses for pleiotropy and heterogeneity, we were able to reduce the risk of pleiotropy and heterogeneity. By performing weighed median estimates and MR Egger regressions we tested for pleiotropic bias. We also searched for known pleiotropic effects and performed Cochran’s Q tests to examine heterogeneity. The sensitivity analyses provided supporting evidence for a causal association between high blood pressure and QRS amplitudes. Another limitation is that for some cardiovascular risk factors (e.g. cigarettes smoked per day and fasting insulin), the instrumental variables do not explain a large proportion of the variation in these traits. Identifying more genetic variants associated with these risk factors will lead to more statistical power to find causal associations between these risk factors and QRS traits, resulting in a lower probability of finding false negative results. Also, in the MR analyses, unmeasured confounders may be involved. Therefore, a limitation of the current study is that we cannot entirely rule out that our analyses are unbiased from any (unmeasured) confounder. The GWAS meta-analyses results used for our MR analyses were based on genetic associations test including possible confounders. For example, the meta-analysis of GWAS on QRS traits was tested including age, gender, height and body mass index as covariates^12^. Additionally, the genetic variants associated with cardiovascular risk factors, QRS traits and mortality and longevity were obtained in predominantly Caucasians, minimizing the possibility of population stratification bias, but also limit our study to Caucasians and our results are therefore not generalizable to other ethnicities. Finally, since we exclusively used genetic variants of published GWAS with complete information on the effect size (β) with standard error (SE) and effect and non-effect allele of each variant, we could not use the most recent GWAS publications for some of the cardiovascular risk factors. Adding more variants to our analyses may have improved the risk predictions and statistical power.

## Conclusion

High blood pressure likely causes larger QRS amplitudes on the ECG. Genetically predicted larger QRS duration and amplitudes in the general population are not linked to longevity, suggesting a more prominent role of acquired factors explaining the phenotypic link between QRS abnormalities and outcome.

## Methods

### Study design

Our study design consisted of two stages. 1) First, we identified genetic variants associated with cardiovascular risk factors (blood pressure, body mass index, lipids and diabetes and smoking) in previously published GWAS data (Supplementary Tables 4–16*)*. 2) We applied these genetic predictors of cardiovascular risk factors (instrumental variables for the exposure traits) to a large-scale GWAS of QRS traits (the Sokolow-Lyon, Cornell, and 12-lead-voltage (12-leadsum) duration products and QRS duration) to determine which cardiovascular risk factors might lead causally to QRS changes (Fig. 1A). This GWAS meta-analysis was performed in up to 73,518 individuals of European ancestry from 24 studies with 12-lead ECG data^12^. The mean age of the participants included in the individual studies of this GWAS meta-analysis ranged from 39 to 76 years. The percentage of women included in the individual studies ranged from 0% to 95%.

Subsequently, we used the genetic variants associated with QRS traits as instrumental variables in MR analyses to determine whether a causal relationship exist between *genetically predicted* QRS traits and mortality and longevity (outcome, Fig. 1B).

### Identification of genetic variants associated with cardiovascular risk factors

Genetic variants genome-wide significantly (P < 5 × 10^−8^) associated with cardiovascular risk factors were obtained from previously published summary statistics of GWAS (Table 2), filtered on P value and clumped on linkage disequilibrium using the MR base R package using default settings (https://mrcieu.github.io/TwoSampleMR/). We exclusively used GWAS datasets with complete information on the effect size (β) with standard error (SE), effect and non-effect allele of each variant and the variance explained (R^2^) by the genetic variants. To ensure the strength of the instruments, we generated F-statistics for these genetic variants (Table 2). The F-statistics were calculated using the formula [R^2^ × (n − 1 − K)]/[(1 − R^2^) × K], in which R^2^ represents the proportion of variability of each cardiovascular risk factor that is explained by the genetic variants, *n* represents sample size, and K represents the number of IVs included in the model. An F-value above ten indicates that a causal estimate is unlikely to be biased due to weak instruments^26^. For each cardiovascular risk factor variant, we determined the effect of these variants on the QRS traits in the abovementioned raw GWAS meta-analysis data^12^. All variants per cardiovascular risk factor and their effects on these risk factors and effect and non-effect allele are listed in Supplementary Tables 4–16. Before MR analyses were performed, the exposure data (variant on risk factor) and outcome data (variant on QRS trait) were harmonized to guarantee that the effect corresponded to the same allele. Summary statistics between exposures and outcomes were harmonized in R using the MR-base package using default settings. Genetic variants were discarded from analysis if alleles did not correspond for the same genetic variant. In case of palindromic genetic variants (if alleles on the forward strand are the same as on the reverse strand), outcome effects were flipped if the alleles on the reverse strand did not correspond to alleles on the forward strand based on the effect allele frequency (if the allele frequency was above 0.42, genetic variants were discarded).

**Table 2:** SNPs associated with cardiovascular risk factors.

Trait	Consortium	IVs (*n)*	Unit	Sample size (*n*)	Mean age range (years)*	Women (% range)**	Ancestry	R^2^ (%)	F-statistics	Pubmed ID
Systolic Blood pressure	74 European cohorts	55	mmHg	201,529	21.5–75.6	0.0–77.2	European	3.4	2588.6	27618452^30^
Diastolic blood pressure	74 European cohorts	57	mmHg	201,529	21.5–75.6	0.0–77.2	European	3.5	2667.5	27618452^30^
HDL cholesterol	GLGC	89	SD	187,167	21.5–75.0	0.0–69.6	European	1.6	1196.4	24097068^31^
LDL cholesterol	GLGC	80	SD	173,082	21.5–75.0	0.0–69.6	European	2.4	1808.8	24097068^31^
Triglycerides	GLGC	54	SD	177,861	21.5–75.0	0.0–69.6	European	2.1	1578.0	24097068^31^
Total cholesterol	GLGC	88	SD	187,365	21.5–75.0	0.0–69.6	European	2.6	1963.5	24097068^31^
Fasting glucose	MAGIC	35	mmol/L	133,010	11.5–75.7	0.0–71.3	European	4.8	3707.8	22885924^32^
Fasting Insulin	MAGIC	14	Log pmol/L	108,557	11.5–75.7	0.0–71.3	European	1.2	893.9	22885924^32^
Body mass index	GIANT	79	SD	339,224	18.9–75.7	0.0–100.0	European	2.7	2041.1	25673413^33^
Waist Hip Ratio adjusted Body mass index	GIANT	31	SD	224,459	18.9–75.3	0.0–100.0	European	1.4	1044.9	25673412^34^
Apolipoprotein A-I	14 European cohorts	11	SD	20,687	23.9–60.9	37.0–60.0	European	5.0	3870.4	27005778^35^
Apolipoprotein B	14 European cohorts	21	SD	20,690	23.9–60.9	37.0–60.0	European	8.6	6918.5	27005778^35^
Cigarettes smoked per day	TAG	1	Cigarettes per day	68,028	39.6–72.3	11.6–100	European	0.5	370.4	20418890^36^

HDL = High Density Lipoprotein; LDL = Low Density Lipoprotein.

*Range of the mean age of the participants included in the individual studies of the GWAS meta-analysis.

**Range of the percentage of women included in the individual studies of the GWAS meta-analysis.

### Mendelian randomization analyses

To determine the effect of cardiovascular risk factors on QRS traits, inverse-variance-weighted (MR-IVW) fixed-effects meta analyses were performed, as described before^11^. The cardiovascular risk factors were considered as the exposures and the QRS traits as the outcomes (Fig. 1A). Units of the cardiovascular risk factors for which MR analyses were scaled are listed in Table 2. For the MR analyses, we considered a multiple testing (Bonferroni-corrected) P-value < 9.62 × 10^−5^ (0.005/(13 risk factors x 4 QRS traits)) as statistically significant^27^.

To determine the effect (β) of genetic variants associated with QRS traits on mortality, MR-IVW analyses were performed in which the QRS traits were considered as exposures and mortality and (parental) longevity as outcomes (Fig. 1B). To determine the effect of the genetic variants associated with QRS traits on mortality, cox regression analyses on mortality with these genetic variants (P < 1 × 10^−8^) were performed in all participants with genetic information of the UK Biobank (N = 143,193). For the analyses of longevity, previously published summary statistics of GWAS on longevity were used as outcome data^28^. Longevity was defined as combined mothers and fathers age at death. For sensitivity purposes, we attempted to increase power of the instruments by adding genetic variants with higher P-values for QRS traits (P < 1 × 10^−7^, <1 × 10^−6^, <1 × 10^−5^ & <1 × 10^−4^) and repeated the MR analyses.

To explore pleiotropy or heterogeneity of our effect estimates, we evaluated weighted median estimate, MR Egger regression and Cochran’s Q test. The weighed median estimate and MR Egger regressions were performed to test for pleiotropic bias. Where the MR-IVW method assumes absence of pleiotropic effects for all included genetic variants, the weighted median estimation allows that up to 50% of the weight of genetic variants comes from invalid instruments^29^. The MR Egger regression method allows all the instrumental variables to be invalid, and is therefore less powerful than the weighted median estimate^29^. The estimated value of the intercept in Egger regression can be interpreted as an estimate of the average pleiotropic effect across the genetic variants, since the intercept will differ from zero when the estimates from small studies are more skewed towards either high or low values compared to large studies^11^. P-value and the intercept (including SE) with the Y-axis were reported. P-values < 0.10 of the Egger’s intercept were considered to provide evidence for pleiotropic bias. In addition, we performed heterogeneity statistics using the Cochran’s Q test and reported the heterogeneity P-value. A heterogeneity P-value < 0.05 was considered to provide evidence of heterogeneity, in which case MR analyses were repeated without the instrumental variables that were individually associated (P < 0.05) with QRS traits. Power calculations were carried out using the online tool http://cnsgenomics.com/shiny/mRnd/. The power could not be calculated directly as different units were used for the different traits and only summary statistics were available. Therefore, we estimated the statistical power for the MR analyses of blood pressure on QRS traits and of QRS traits on mortality and longevity using standardized values. These standardizes values (z-scores) were calculated using the mean and standard deviations of these variables. In the UK Biobank dataset, the minimum detectable odds ratio between genetically predicted QRS traits and mortality was estimated at 1.25 per standard deviation of the QRS traits. The minimum detectable effect for an association between genetically predicted QRS traits and longevity (in years) was estimated at β = 0.1 per standard deviation of the QRS traits.

Forest plots were constructed for adjusted effects of instrumental variables on QRS duration and amplitude if specific cardiovascular risk factors were associated with QRS traits. Scatterplots and trend lines (MR Egger and IVW) were plotted to provide insights into the individual instrumental variable effects on QRS duration and amplitude compared to the effects sizes of the cardiovascular risk factors. All MR analyses were performed using R version 3.3.2.

### Data availability

The genetic data associated with cardiovascular risk factors analysed during this study are included in this published article and its Supplementary Information files. The genetic data associated with QRS traits or longevity are available as summary statistics online. The dataset of mortality is available from the corresponding author on reasonable request.

## Electronic supplementary material

Supplementary material
