# Author Correction: Causal Pathways from Blood Pressure to Larger QRS Amplitudes: a Mendelian Randomization Study

**DOI:** 10.1038/s41598-018-27848-6

**Published:** 2018-07-03

**Authors:** M. Yldau Van Der Ende, Tom Hendriks, Dirk J. Van Veldhuisen, Harold Snieder, Niek Verweij, Pim Van Der Harst

**Affiliations:** 1 University of Groningen, University Medical Center Groningen, The department of Cardiology, Groningen, The Netherlands; 2 University of Groningen, University Medical Center Groningen, The department of Epidemiology, Groningen, The Netherlands

Correction to: *Scientific Reports* 10.1038/s41598-018-24002-0, published online 11 April 2018

The original version of this Article contained typographical errors in title of the paper, where ‘QRS’ was incorrectly given as “Qrs” and a colon was omitted. This has now been corrected in the PDF and HTML versions of the Article and in the accompanying Supplementary Information file.

