# Supplementary material for: Causal Pathways from Blood Pressure to Larger QRS Amplitudes: a Mendelian Randomization Study
Source: Sci Rep. 2018 Apr 11;8:5817. doi: 10.1038/s41598-018-24002-0 (PMC5895613; doi:10.1038/s41598-018-24002-0)
Supplement: Supplementary file 1 — Supplementary material [file 41598_2018_24002_MOESM1_ESM.pdf]

Causal Pathways from Blood Pressure to Larger QRS Amplitudes: a Mendelian Randomization Study

M. Yldau VAN DER ENDE BSc[a], Tom HENDRIKS M.D.[a] , Dirk J. VAN VELDHUISEN, M.D., PhD.[a], Harold SNIEDER, Ph.D.[b], Niek VERWEIJ, Ph.D.[a], Pim VAN DER HARST, M.D., Ph.D.[a]

Author Affiliations: [a]From the University of Groningen, University Medical Center Groningen, The department of Cardiology, Groningen, The Netherlands. [b]From the University of Groningen, University Medical Center Groningen, The department of Epidemiology, Groningen, The Netherlands.

Supplementary Figures 1-5

Supplementary Tables 1-16

Supplementary Figure 1

Sensitivity analyses: Forest plot and Scatter plot of SBP on Cornell product. Instrumental variables independently associated with Cornell product were removed from analyses.

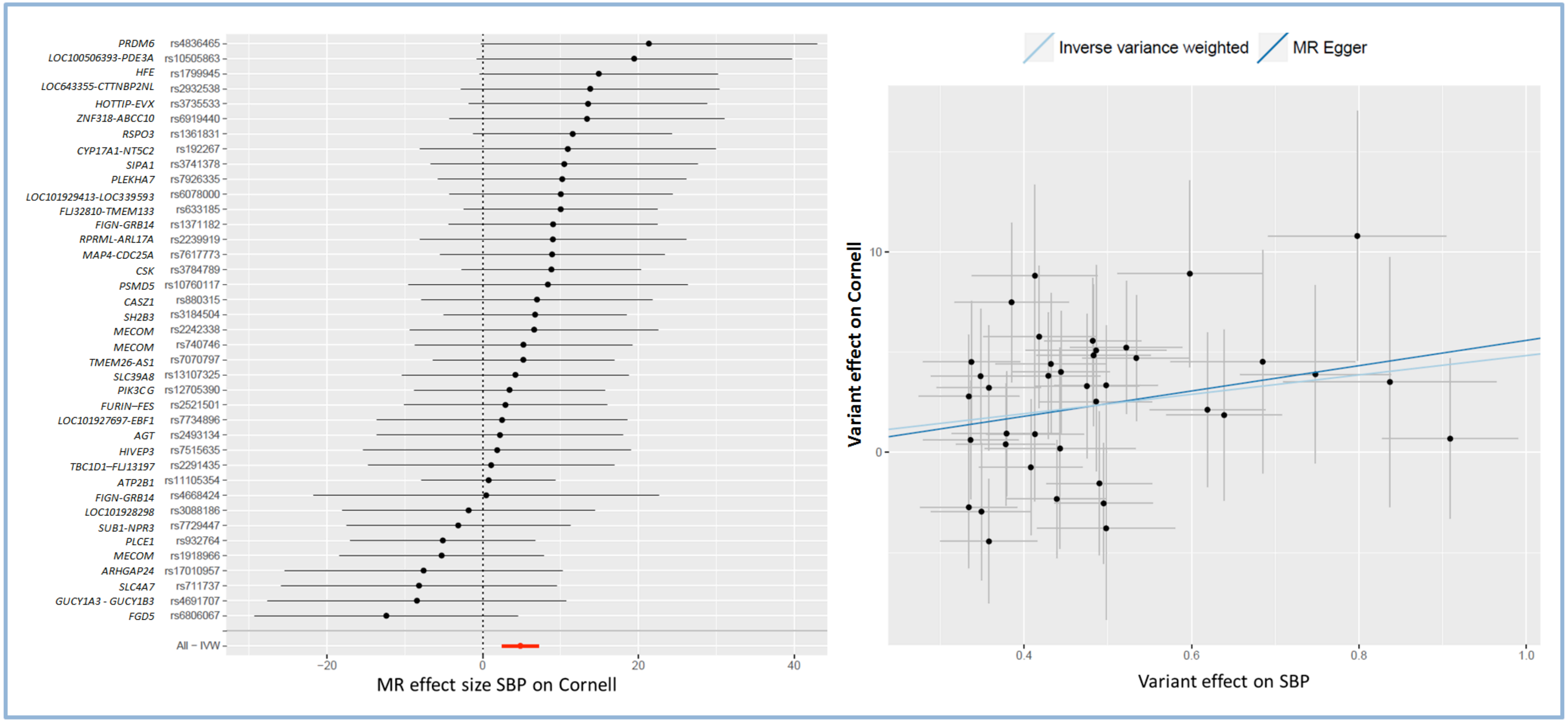

Left: In the forest plot the Mendelian Randomization effect size of systolic blood pressure on Cornell product is displayed on the X-axis. On de Y-axis the different genetic variants were listed. **Right**: In the scatterplot the variant effects on systolic blood pressure is displayed on the X-axis and the variant effect on Cornell product on the Y-axis. The light blue line is the regression line of the inverse-variance-weighted fixed-effects meta analyses. The dark blue line is the regression line of the MR Egger regression line. MR = mendelian randomization, SBP = Systolic blood pressure.

Supplementary Figure 2

Sensitivity analyses: Forest plots of DBP and SBP on Sokolow-Lyon product. Instrumental variables independently associated with Sokolow-Lyon were removed from analyses.

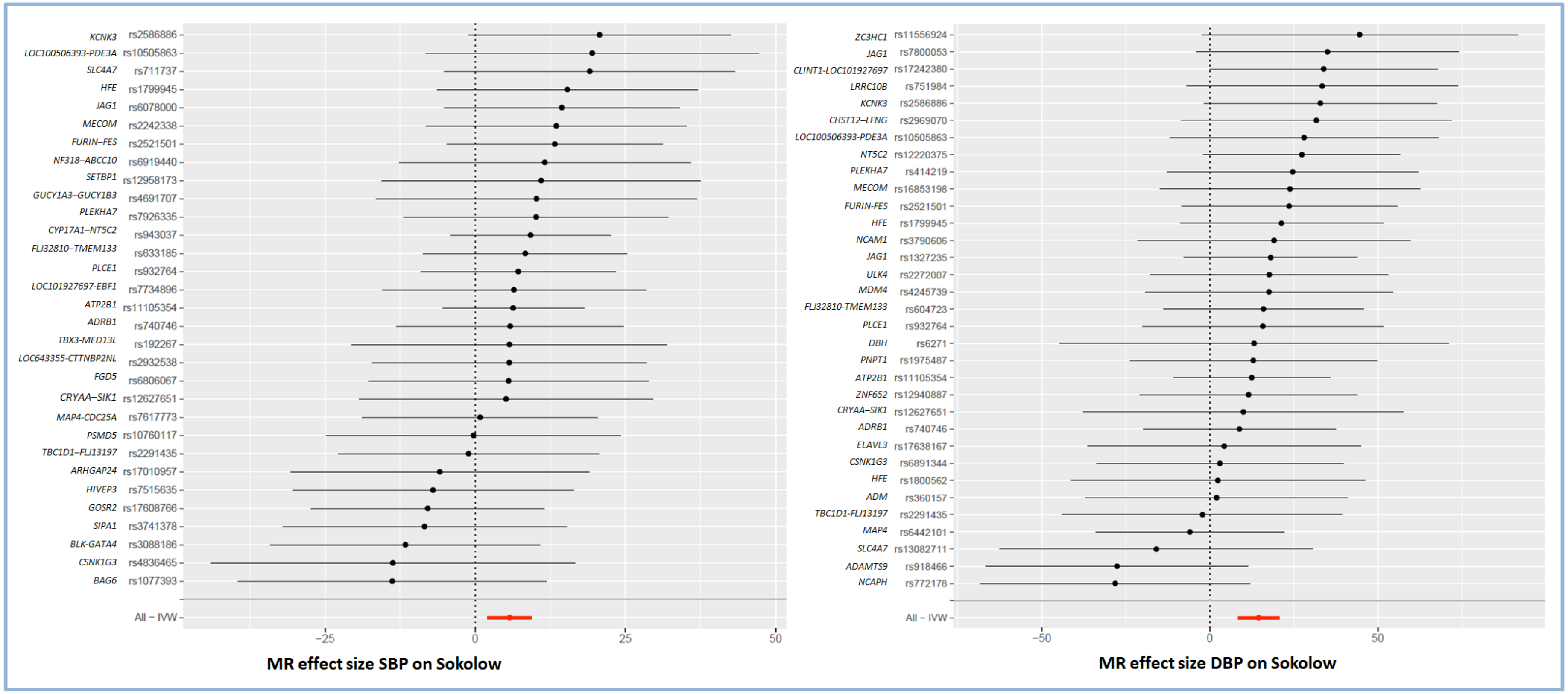

On the X-axis the Mendelian Randomization effect size of blood pressure on Sokolow-Lyon product were displayed. On de Y-axis the different genetic variants were listed. DBP = diastolic blood pressure, MR = mendelian randomization, SBP = systolic blood pressure.

Supplementary Figure 3

Sensitivity analyses: Scatter plots of DBP and SBP on Sokolow-Lyon product. Instrumental variables independently associated with Sokolow-Lyon were removed from analyses.

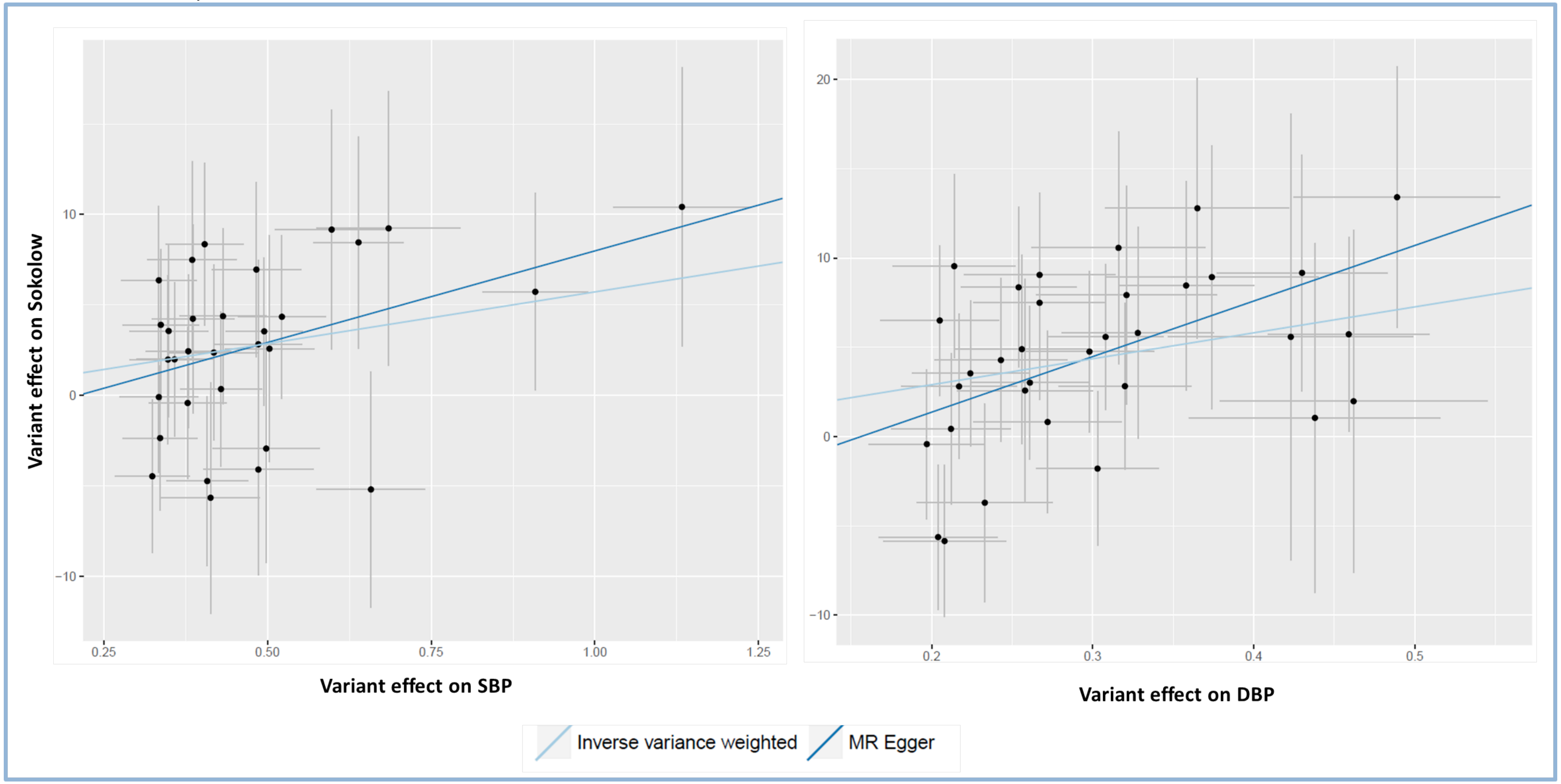

On the X-axis the variant effects on blood pressure are displayed and on the Y-axis the variant effect on Sokolow-Lyon product. The light blue line is the regression line of the inverse-variance-weighted fixed-effects meta analyses. The dark blue line is the regression line of the MR Egger regression line. DBP = diastolic blood pressure, MR = mendelian randomization, SBP = systolic blood pressure

Supplementary Figure 4

Sensitivity analyses: Forest plots of DBP and SBP on Lead sum product. Instrumental variables independently associated with Lead Sum were removed from analyses.

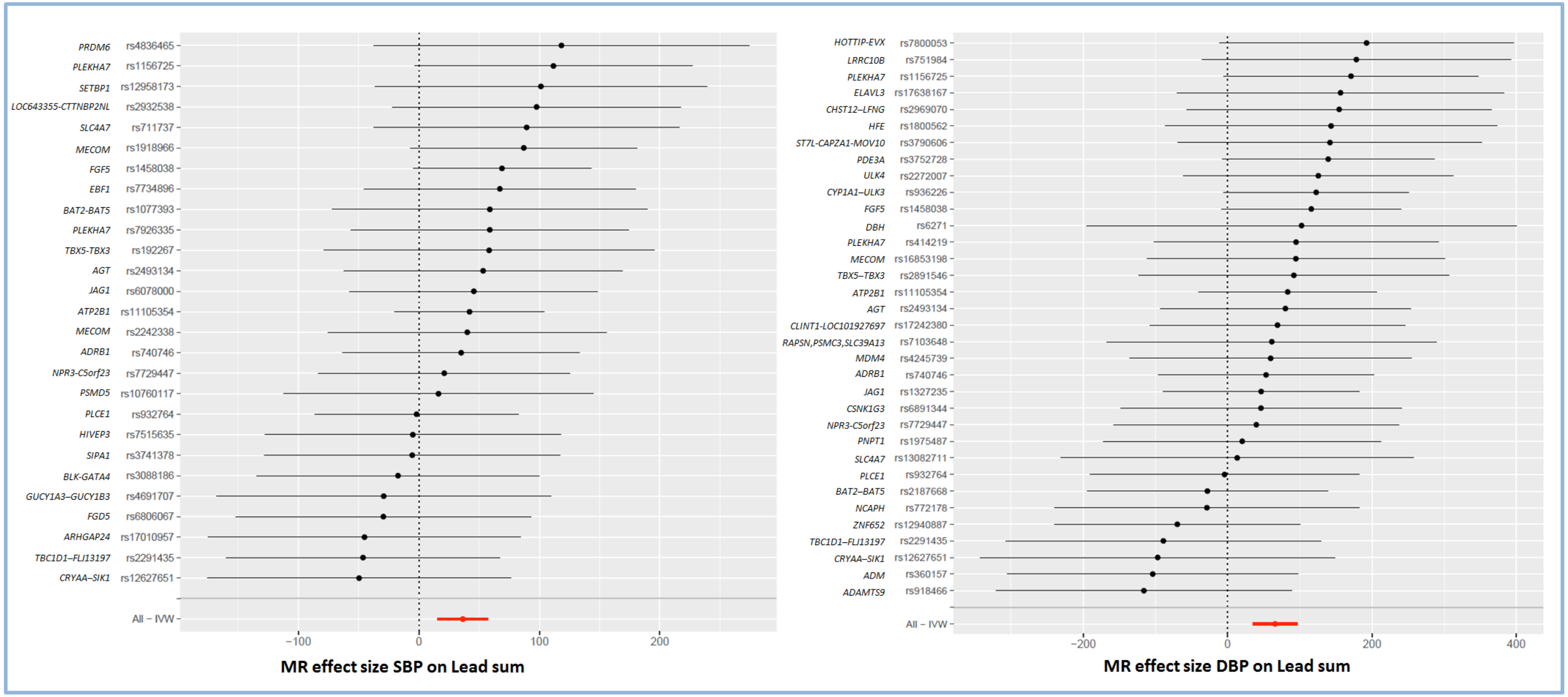

On the X-axis the Mendelian Randomization effect size of blood pressure on Lead sum product were displayed. On de Y-axis the different genetic variants were listed. DBP = diastolic blood pressure, MR = mendelian randomization, SBP = systolic blood pressure

Supplementary Figure 5

Sensitivity analyses: Scatter plots of DBP and SBP on Lead sum product. Instrumental variables independently associated with Lead Sum were removed from analyses.

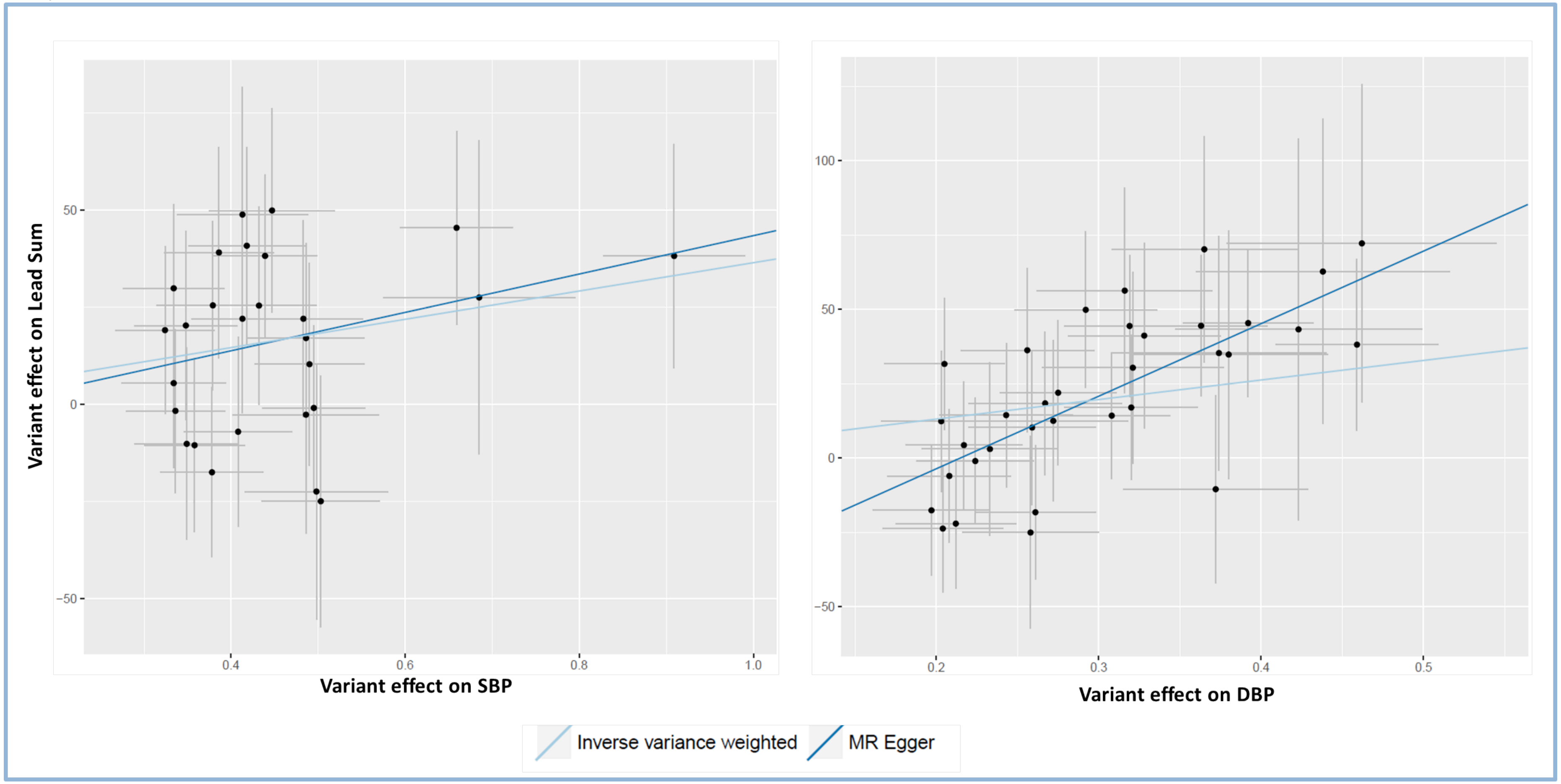

On the X-axis the variant effects on blood pressure are displayed and on the Y-axis the variant effect on Lead sum product. The light blue line is the regression line of the inverse-variance-weighted fixed-effects meta analyses. The dark blue line is the regression line of the MR Egger regression line. DBP = diastolic blood pressure, MR = mendelian randomization, SBP = systolic blood pressure.

Supplementay Table 1: MR analyses cardiovascular risk factors on QRS traits

Risk factor	QRS trait	P-value	β (SE)	MR Egger intercept	P-value	Heterogeneity P-value
**HDL**	**QRS duration**	0.945				
	Sokolow-Lyon	0.558				
	Cornell	0.638				
	12-lead sum	0.187				
**LDL**	QRS duration	0.249				
	Sokolow-Lyon	0.453				
	Cornell	0.407				
	12-lead sum	0.395				
**Triglyceride**	QRS duration	0.407				
	Sokolow-Lyon	0.635				
	Cornell	0.162				
	12-lead sum	0.96				
**Total Cholesterol**	QRS duration	0.763				
	Sokolow-Lyon	0.684				
	Cornell	0.262				
	12-lead sum	0.344				
**Fasting Glucose**	QRS duration	0.601				
	Sokolow-Lyon	0.416				
	Cornell	0.814				
	12-lead sum	0.587				
**Fasting Insulin**	QRS duration	0.371				
	Sokolow-Lyon	0.489				
	Cornell	0.465				
	12-lead sum	0.747				
**BMI**	QRS duration	0.619				
	Sokolow-Lyon	0.372				
	Cornell	0.514				
	12-lead sum	0.29				
**Waist Hip Ratio adjusted BMI**	QRS duration	0.658				
	Sokolow-Lyon	0.179				
	Cornell	0.113				
	12-lead sum	0.326				
**Apo A**	QRS duration	0.551				
	Sokolow-Lyon	0.454				
	Cornell	0.388				
	12-lead sum	0.781				
**Apo B**	QRS duration	0.292				
	Sokolow-Lyon	0.917				
	Cornell	0.176				
	12-lead sum	0.621				
**Cigarettes Per day**	QRS duration	0.509				
	Sokolow-Lyon	0.358				
	Cornell	0.369				
	12-lead sum	0.274				

Apo A = Apolipoprotein A; Apo B = Apolipoprotein B; β = beta; BMI = Body Mass Index; HDL = High Density Lipoprotein; LDL = Low Density Lipoprotein; SE = Standard Error

Supplementay Table 2: Sensitivity analyses: MR analyses blood pressure on QRS traits without individually significant IVs on QRS traits

Risk factor	QRS trait	IVs (N)	P-value	β (SE)	MR Egger intercept	P-value	Heterogeneity P-value
**SBP**	Sokolow-Lyon	31	**2.56×10^{-3}**	5.72 (1.90)	-2.14 (2.90)	0.466	0.936
	Cornell	39	**5.19×10^{-5}**	4.83 (1.19)	-0.72 (2.23)	0.748	0.750
	12-lead sum	27	**6.38×10^{-4}**	36.45 (10.67)	-6.00 (18.77)	0.752	0.904
**DBP**	Sokolow-Lyon	33	**3.71×10^{-6}**	14.52 (3.14)	-4.86 (3.56)	0.182	0.883
	12-lead sum	34	**3.75×10^{-5}**	65.74 (15.95)	-52.35 (18.94)	**9.38×10^{-3}**	0.890

β = beta, DBP = diastolic blood pressure, IV = instrumental variables, MR = Mendelian Randomization, SBP= systolic blood pressure, SE = Standard error

Supplementay Table 3: Sensitivity analyses by adding more IVs with higher P-values did not show any associations with mortality or longevity.

QRS Trait	P value IVS	IVs (N)	P-Values: All-cause Mortality	CV Mortality	Fathers age at Death	Mothers age at Death
**QRS duration**	**< $1x10^{-8}$**	31	0.376	0.283	0.477	0.52
	**< $1x10^{-7}$**	40	0.728	0.422	0.669	0.553
	**< $1x10^{-6}$**	54	0.592	0.277	0.547	0.4
	**< $1x10^{-5}$**	83	0.929	0.496	0.597	0.396
	**< $1x10^{-4}$**	215	0.81	0.839	0.177	0.366
**Sokolow-Lyon**	**< $1x10^{-8}$**	15	0.509	0.765	-	0.842
	**< $1x10^{-7}$**	22	0.362	0.496	0.93	0.357
	**< $1x10^{-6}$**	31	0.388	0.647	0.482	0.391
	**< $1x10^{-5}$**	71	0.472	0.942	0.559	0.386
	**< $1x10^{-4}$**	220	0.833	0.69	0.557	0.022
**Cornell**	**< $1x10^{-8}$**	16	0.845	0.692	0.719	0.32
	**< $1x10^{-7}$**	21	0.544	0.406	0.526	0.695
	**< $1x10^{-6}$**	29	0.556	0.536	0.64	0.665
	**< $1x10^{-5}$**	64	0.9	0.528	0.766	0.659
	**< $1x10^{-4}$**	212	0.916	0.712	0.571	0.265
**Lead Sum**	**< $1x10^{-8}$**	26	0.396	0.119	0.756	0.94
	**< $1x10^{-7}$**	36	0.684	0.266	0.197	0.602
	**< $1x10^{-6}$**	48	0.495	0.16	0.17	0.317
	**< $1x10^{-5}$**	72	0.268	0.186	0.19	0.254
	**< $1x10^{-4}$**	238	0.842	0.862	0.928	0.904

CV = Cardiovascular, IV = instrumental variables

Supplementay Table 4: Genetic variants associated with systolic blood pressure

rsID	EFAL	NEFAL	EAF	B	SE	P-value
rs11105354	A	G	0.84	0.909	0.081	3.88E-29
rs17037390	G	A	0.845	0.908	0.081	5.95E-29
rs943037	C	T	0.913	1.133	0.105	2.35E-27
rs1458038	T	C	0.3	0.659	0.065	5.36E-24
rs6026748	A	G	0.125	0.867	0.089	3.15E-22
rs2521501	T	A	0.316	0.639	0.069	3.35E-20
rs12705390	A	G	0.227	0.619	0.069	2.69E-19
rs932764	G	A	0.446	0.495	0.059	6.88E-17
rs1361831	C	T	0.459	0.482	0.058	7.38E-17
rs3784789	C	G	0.352	0.534	0.064	1.01E-16
rs7070797	G	A	0.87	0.748	0.09	1.14E-16
rs12656497	C	T	0.597	0.487	0.06	3.85E-16
rs3184504	T	C	0.475	0.498	0.062	9.97E-16
rs17608766	C	T	0.146	0.658	0.083	2.27E-15
rs633185	C	G	0.715	0.522	0.067	6.97E-15
rs7729447	G	A	0.679	0.49	0.063	7.2E-15
rs1371182	C	T	0.557	0.444	0.058	1.89E-14
rs592373	A	G	0.64	0.484	0.063	2.02E-14
rs880315	C	T	0.359	0.475	0.062	2.09E-14
rs3735533	C	T	0.92	0.798	0.106	6.48E-14
rs1918966	A	G	0.461	0.439	0.06	1.89E-13
rs12627651	A	G	0.292	0.503	0.068	1.97E-13
rs740746	A	G	0.73	0.486	0.067	4.59E-13
rs6078000	G	A	0.273	0.483	0.068	9.39E-13
rs2493134	C	T	0.421	0.413	0.058	9.65E-13
rs1620668	G	A	0.178	0.535	0.076	1.45E-12
rs1799945	G	C	0.143	0.598	0.086	3.28E-12
rs1450271	T	C	0.468	0.413	0.059	3.4E-12
rs7617773	T	C	0.684	0.429	0.062	5.31E-12
rs2586886	C	T	0.401	0.404	0.059	5.94E-12
rs11014171	C	T	0.66	0.419	0.061	6.5E-12
rs13107325	C	T	0.93	0.837	0.127	4.69E-11
rs3088186	T	C	0.309	0.408	0.062	4.94E-11
rs7926335	T	C	0.264	0.432	0.066	7.67E-11
rs2291435	C	T	0.487	0.378	0.059	1.03E-10
rs2932538	G	A	0.747	0.418	0.067	3.79E-10
rs2242338	A	C	0.923	0.685	0.11	5.55E-10
rs1156725	C	T	0.196	0.447	0.072	5.65E-10
rs6806067	A	C	0.444	0.358	0.058	7.19E-10
rs12958173	A	C	0.313	0.386	0.063	1.19E-09
rs17010957	C	T	0.143	0.498	0.082	1.51E-09
rs192267	G	T	0.474	0.348	0.059	3.2E-09
rs7515635	T	C	0.474	0.336	0.057	4.54E-09
rs6919440	G	A	0.43	0.337	0.058	4.92E-09
rs7734896	G	T	0.559	0.379	0.065	6.5E-09
rs4691707	G	A	0.348	0.349	0.06	7.1E-09
rs2239919	A	G	0.351	0.358	0.062	7.39E-09
rs3741378	C	T	0.863	0.486	0.084	8.04E-09
rs12247028	G	A	0.389	0.364	0.063	8.16E-09
rs711737	A	C	0.604	0.334	0.058	9.93E-09
rs10505863	C	T	0.742	0.385	0.068	1.24E-08
rs1077393	G	A	0.471	0.324	0.057	1.64E-08
rs2594992	C	A	0.393	0.334	0.06	2.31E-08
rs10760117	T	G	0.414	0.334	0.06	2.54E-08

rs4836465	G	A	0.184	0.413	0.075	3.83E-08

Supplementay Table 5: Genetic variants associated with diastolic blood pressure

rsID	EFAL	NEFAL	EAF	B	SE	P-value
rs6026748	A	G	0.126	0.552	0.055	4.86E-24
rs17037390	G	A	0.845	0.499	0.05	1.2E-23
rs1458038	T	C	0.302	0.392	0.04	7.36E-23
rs3184504	T	C	0.473	0.362	0.038	1.28E-21
rs11105354	A	G	0.841	0.459	0.05	2.61E-20
rs12243859	C	T	0.676	0.335	0.038	8.11E-19
rs936226	C	T	0.278	0.363	0.041	1.03E-18
rs7076398	T	A	0.812	0.409	0.047	2.55E-18
rs1327235	G	A	0.458	0.308	0.036	1.78E-17
rs2521501	T	A	0.316	0.358	0.042	1.85E-17
rs1799945	G	C	0.142	0.43	0.053	3.1E-16
rs6442101	T	C	0.693	0.303	0.038	1.6E-15
rs3752728	A	G	0.738	0.319	0.04	2.35E-15
rs740746	A	G	0.73	0.32	0.041	8.63E-15
rs2493134	C	T	0.421	0.275	0.036	9.53E-15
rs13190766	G	A	0.458	0.271	0.035	1.42E-14
rs13107325	C	T	0.93	0.602	0.078	1.63E-14
rs12220375	T	C	0.912	0.489	0.064	2.23E-14
rs604723	C	T	0.724	0.298	0.04	1.02E-13
rs592373	A	G	0.64	0.282	0.039	3.61E-13
rs12940887	T	C	0.381	0.261	0.037	1.07E-12
rs9287843	C	T	0.554	0.254	0.036	1.13E-12
rs2586886	C	T	0.402	0.254	0.036	1.92E-12
rs2272007	T	C	0.18	0.328	0.047	3.94E-12
rs929250	T	G	0.919	0.452	0.066	6.4E-12
rs880315	C	T	0.36	0.257	0.038	1.34E-11
rs7729447	G	A	0.679	0.259	0.039	1.88E-11
rs1156725	C	T	0.196	0.292	0.044	3.67E-11
rs4247374	C	T	0.857	0.369	0.056	3.89E-11
rs2187668	C	T	0.874	0.372	0.057	4.31E-11
rs223102	C	T	0.468	0.235	0.036	6.4E-11
rs10505863	C	T	0.742	0.267	0.041	1.07E-10
rs7800053	G	A	0.112	0.365	0.057	1.82E-10
rs2891546	G	A	0.89	0.38	0.061	4.71E-10
rs3790606	C	G	0.35	0.256	0.041	5.15E-10
rs12627651	A	G	0.292	0.258	0.042	6.24E-10
rs932764	G	A	0.448	0.224	0.036	6.28E-10
rs1975487	G	A	0.544	0.217	0.036	1.27E-09
rs17080093	C	T	0.925	0.411	0.068	1.71E-09
rs6891344	A	G	0.818	0.272	0.046	3.06E-09
rs4245739	A	C	0.739	0.243	0.041	4.63E-09
rs751984	T	C	0.872	0.316	0.054	4.92E-09
rs360157	T	C	0.598	0.212	0.037	7.18E-09
rs414219	G	C	0.134	0.321	0.056	7.58E-09
rs17242380	C	T	0.254	0.267	0.047	9.28E-09
rs16853198	A	G	0.92	0.374	0.066	1.31E-08
rs11556924	C	T	0.622	0.214	0.038	1.76E-08
rs17638167	C	T	0.95	0.462	0.083	0.000000022
rs1800562	A	G	0.061	0.438	0.078	2.39E-08
rs6271	C	T	0.93	0.423	0.076	2.61E-08
rs918466	G	A	0.597	0.204	0.037	0.000000027
rs2969070	G	A	0.354	0.205	0.037	3.21E-08
rs772178	G	A	0.363	0.208	0.038	3.58E-08
rs13082711	C	T	0.227	0.233	0.042	4.05E-08

rs7103648	G	A	0.383	0.203	0.037	4.39E-08
rs2291435	C	T	0.488	0.197	0.036	4.43E-08
rs4612801	C	T	0.494	0.197	0.036	4.53E-08

Supplementay Table 6: Genetic variants associated with high density lipoprotein

rsID	EFAL	NEFAL	EAF	B	SE	P-value
rs9989419	G	A	0.595	0.1473	0.0036	1E-200
rs10468017	T	C	0.2757	0.1179	0.0038	1.21E-188
rs13702	C	T	0.3127	0.1058	0.0038	1.28E-160
rs4939883	C	T	0.8193	0.0799	0.0045	1.796E-66
rs1883025	C	T	0.7573	0.0698	0.0041	1.496E-65
rs2241770	T	C	0.8971	0.0989	0.0057	6.784E-60
rs633695	G	A	0.285	0.0885	0.0054	7.82E-58
rs676210	A	G	0.2309	0.066	0.004	2.345E-54
rs16942887	A	G	0.1332	0.0831	0.0051	8.281E-54
rs964184	C	G	NA	0.1065	0.0071	6.086E-48
rs4240624	A	G	0.9248	0.0818	0.0058	1.323E-45
rs4846914	A	G	0.5844	0.0479	0.0034	3.513E-41
rs4465830	A	G	0.7982	0.0597	0.0044	5.175E-40
rs3847502	A	C	0.314	0.048	0.0036	3.306E-38
rs838876	A	G	0.3259	0.0493	0.0039	7.325E-33
rs103294	T	C	0.186	0.0523	0.0044	3.995E-30
rs10808546	T	C	0.4459	0.0409	0.0034	4.106E-30
rs102275	T	C	0.628	0.0391	0.0035	6.404E-28
rs686030	A	C	0.8588	0.055	0.0049	4.292E-27
rs2075650	A	G	0.8734	0.0554	0.0051	9.716E-26
rs1689797	C	A	0.6979	0.0358	0.0036	2.852E-21
rs11789603	T	C	0.08971	0.06	0.006	3.695E-21
rs2241210	G	A	0.5528	0.0332	0.0035	2.489E-20
rs1877031	A	G	0.6755	0.0336	0.0036	1.204E-19
rs2925979	C	T	0.7045	0.0351	0.0037	1.321E-19
rs7306660	G	A	0.6306	0.0345	0.0036	3.341E-19
rs4660293	A	G	0.7639	0.0353	0.004	2.863E-18
rs2278236	A	G	0.5435	0.0331	0.0035	3.185E-18
rs1515110	G	T	0.3813	0.0323	0.0035	8.038E-18
rs181360	T	G	0.8008	0.0376	0.0042	9.238E-18
rs2250802	G	A	0.3193	0.034	0.0038	2.022E-17
rs11765979	C	A	0.4578	0.0412	0.0048	3.111E-17
rs2293889	G	T	0.5871	0.0312	0.0035	4.271E-17
rs737337	T	C	0.9314	0.0565	0.0061	4.564E-17
rs12748152	C	T	0.92876	0.0506	0.0062	9.737E-16
rs13107325	C	T	0.92216	0.0708	0.0078	1.065E-15
rs12740374	T	G	0.2124	0.0343	0.0041	1.687E-15
rs7607980	C	T	0.1491	0.0447	0.0052	1.807E-15
rs2288912	G	C	0.4987	0.0297	0.0036	7.148E-15
rs2454722	G	A	0.1451	0.0351	0.0044	3.308E-14
rs4148005	T	G	0.7005	0.0283	0.0036	5.743E-14
rs3741414	T	C	0.1913	0.0296	0.004	6.095E-14
rs2642438	G	A	0.7454	0.0303	0.0039	7.781E-14
rs205262	A	G	0.7335	0.0283	0.0039	3.878E-13
rs17145738	T	C	0.1174	0.0408	0.0053	4.952E-13
rs687339	C	T	0.2335	0.0316	0.0042	7.108E-13
rs9457931	A	G	0.9314	0.0552	0.0073	7.297E-13
rs492571	T	C	0.95778	0.0663	0.009	1.265E-12
rs4969178	G	A	0.6266	0.0263	0.0035	1.532E-12
rs3822072	G	A	0.5119	0.0251	0.0034	4.058E-12
rs702485	G	A	0.4499	0.0243	0.0034	6.45E-12
rs2013208	T	C	0.5053	0.0254	0.0036	8.916E-12
rs4142995	G	T	0.6161	0.0263	0.0037	9.365E-12
rs4379922	C	T	0.3496	0.0247	0.0036	9.559E-12

rs998584	C	A	0.4855	0.026	0.0038	2.269E-11
rs12133576	A	G	0.3549	0.0243	0.0035	6.15E-11
rs3861397	A	G	0.6583	0.024	0.0036	8.401E-11
rs970548	C	A	0.277	0.0258	0.0039	1.706E-10
rs7112577	G	C	NA	0.0826	0.0129	2.34E-10
rs1936800	C	T	0.5277	0.02	0.0034	3.055E-10
rs1980493	T	C	0.8773	0.0318	0.0048	3.764E-10
rs6450176	G	A	0.7216	0.0254	0.0039	6.875E-10
rs2066714	C	T	0.1201	0.0453	0.0071	7.258E-10
rs1866956	T	C	0.6755	0.0217	0.0037	7.964E-10
rs1047891	C	A	0.6979	0.0269	0.0039	8.73E-10
rs11065987	A	G	0.5778	0.0222	0.0035	1.225E-09
rs12412743	C	T	0.847	0.0291	0.0045	1.307E-09
rs6031587	C	T	0.9314	0.0488	0.0074	1.919E-09
rs10087900	G	A	0.5607	0.0231	0.0036	2.174E-09
rs6567160	T	C	0.7691	0.0257	0.0041	2.918E-09
rs11045163	G	A	0.4063	0.0217	0.0035	3.196E-09
rs731839	A	G	0.6583	0.022	0.0037	3.441E-09
rs2290547	G	A	0.7889	0.0297	0.0046	3.69E-09
rs10761771	C	T	0.467	0.0198	0.0034	4.12E-09
rs13076253	A	C	0.8522	0.0283	0.0048	4.961E-09
rs4650994	G	A	0.5172	0.021	0.0034	6.696E-09
rs16965220	A	C	0.2982	0.0219	0.0037	7.909E-09
rs4983559	G	A	0.3773	0.0197	0.0036	9.565E-09
rs4917014	G	T	0.3404	0.0222	0.0036	1.026E-08
rs499974	C	A	0.8245	0.0263	0.0044	1.12E-08
rs6805251	T	C	0.3813	0.02	0.0035	1.332E-08
rs12145743	G	T	0.3311	0.0203	0.0036	1.803E-08
rs13099479	A	G	0.08971	0.036	0.0062	1.819E-08
rs17173637	T	C	0.90237	0.0363	0.0057	1.899E-08
rs12801636	A	G	0.2243	0.0235	0.0042	3.147E-08
rs2606736	C	T	0.3945	0.0246	0.0043	4.799E-08
rs424346	T	C	0.04881	0.0679	0.0113	4.84E-08
rs10019888	A	G	0.8364	0.027	0.0046	4.901E-08
rs2602836	A	G	0.4274	0.0192	0.0034	4.964E-08

rsID	EFAL	NEFAL	EAF	B	SE	P-value
rs7254892	G	A	0.96834	0.4853	0.0119	1E-200
rs646776	T	C	0.7876	0.1602	0.0044	1E-200
rs6511720	G	T	0.90237	0.2209	0.0061	1E-200
rs1367117	A	G	0.2876	0.1186	0.004	9.48E-183
rs11591147	G	T	0.98285	0.497	0.018	8.58E-143
rs12721109	G	A	0.98285	0.4462	0.0183	2.99E-122
rs6544713	T	C	0.2942	0.0806	0.0041	4.843E-83
rs12916	C	T	0.4314	0.0733	0.0038	7.792E-78
rs2965157	T	C	0.97889	0.1886	0.0112	7.292E-62
rs2954029	A	T	0.5317	0.0564	0.0036	2.095E-50
rs579459	C	T	0.215	0.0665	0.0045	2.419E-44
rs2228603	C	T	0.92876	0.104	0.0072	4.433E-44
rs2000999	A	G	0.1847	0.065	0.0046	4.219E-41
rs174583	C	T	0.6253	0.0522	0.0038	7.003E-41
rs247616	C	T	0.7071	0.0547	0.0041	2.566E-37
rs7551981	T	G	0.595	0.0472	0.0038	1.362E-33
rs6882076	C	T	0.6662	0.0456	0.0038	3.31E-31
rs6065311	C	T	0.4604	0.0417	0.0036	1.656E-30
rs964184	G	C	NA	0.0855	0.0078	2.008E-26
rs2587534	A	G	0.5277	0.0391	0.0037	8.055E-25
rs75687619	T	G	0.02375	0.1735	0.0161	8.052E-24
rs9987289	G	A	0.9248	0.0714	0.0066	8.529E-24
rs2073547	G	A	0.1939	0.0485	0.0049	1.923E-21
rs1564348	C	T	0.1451	0.0481	0.005	2.762E-21
rs10893499	A	G	0.1438	0.0521	0.0053	3.861E-21
rs1169288	C	A	0.3338	0.0375	0.004	6.447E-21
rs2738459	A	C	0.5554	0.0532	0.0058	2.261E-19
rs6016373	A	G	0.6266	0.0349	0.0037	7.947E-19
rs10947332	A	G	0.1319	0.0504	0.0056	6.969E-18
rs3757354	C	T	0.7902	0.0382	0.0044	2.087E-17
rs7832643	T	G	0.405	0.0339	0.0038	2.671E-17
rs10903129	G	A	0.5369	0.0328	0.0037	3.03E-17
rs13277801	C	T	0.347	0.0338	0.0038	3.994E-17
rs2642438	G	A	0.7454	0.0352	0.0042	7.318E-16
rs8017377	A	G	0.4591	0.0303	0.0038	2.516E-15
rs4722551	C	T	0.1702	0.0391	0.0049	3.949E-14
rs10832962	T	C	0.719	0.032	0.004	6.619E-14
rs2737252	G	A	0.7441	0.0314	0.0041	7.039E-14
rs2419604	A	G	0.3179	0.0302	0.004	7.49E-14
rs4970712	C	A	0.8061	0.0339	0.0044	2.458E-13
rs6504872	T	C	0.4723	0.0274	0.0037	3.479E-13
rs10490626	G	A	0.92084	0.0508	0.0069	1.697E-12
rs12748152	T	C	0.07124	0.0499	0.0066	3.209E-12
rs4530754	A	G	0.5818	0.0275	0.0036	3.576E-12
rs3184504	C	T	0.5343	0.0268	0.0038	4.203E-12
rs2315065	A	C	0.08707	0.1102	0.0158	5.23E-12
rs16831243	T	C	0.1807	0.0378	0.0055	9.063E-12
rs72902576	T	G	0.96306	0.0933	0.0133	9.576E-12
rs1801689	C	A	0.03694	0.1028	0.0139	9.809E-12
rs7534572	G	C	0.69	0.0407	0.0058	1.288E-11
rs676388	C	T	0.4631	0.0265	0.0039	1.31E-11
rs9875338	G	A	0.6121	0.027	0.0037	2.21E-11
rs4942486	T	C	0.4617	0.0243	0.0037	2.261E-11
rs2886232	T	C	0.1201	0.0451	0.0064	3.876E-11

rs1883025	C	T	0.7573	0.0296	0.0044	6.141E-11
rs6909746	C	T	0.6082	0.0263	0.0037	7.86E-11
rs314253	T	C	0.6649	0.0242	0.0038	3.436E-10
rs364585	G	A	0.6332	0.0249	0.0038	4.278E-10
rs6709904	A	G	0.8865	0.055	0.0085	4.577E-10
rs1800961	C	T	0.9657	0.0685	0.0106	6.034E-10
rs112201728	T	C	0.05805	0.0675	0.0104	8.514E-10
rs3780181	A	G	0.94723	0.0445	0.0074	1.764E-09
rs17404153	G	T	0.8562	0.0336	0.0054	1.832E-09
rs1408272	T	G	0.94723	0.052	0.0083	3.675E-09
rs267733	A	G	0.8628	0.0331	0.0053	5.285E-09
rs2328223	C	A	0.2493	0.0299	0.005	5.632E-09
rs2710642	A	G	0.6187	0.0239	0.0038	6.089E-09
rs16891156	C	A	0.01847	0.0965	0.0171	8.233E-09
rs2030746	T	C	0.3984	0.0214	0.0038	8.605E-09
rs7640978	C	T	0.8945	0.0392	0.0069	9.837E-09
rs12066643	C	T	0.8813	0.0389	0.0064	1.063E-08
rs5763662	T	C	0.02507	0.0767	0.0121	1.191E-08
rs6818397	T	G	0.4129	0.0224	0.004	1.677E-08
rs2390536	A	G	0.3681	0.0223	0.0038	2.037E-08
rs1250229	C	T	0.7889	0.0243	0.0042	3.13E-08
rs4253776	G	A	0.124	0.0311	0.0059	3.353E-08
rs2495495	T	C	0.1346	0.0342	0.0059	3.52E-08
rs10195252	T	C	0.5818	0.0238	0.0039	3.812E-08
rs11563251	T	C	0.1253	0.0345	0.0062	4.499E-08
rs13206249	G	A	0.7836	0.0378	0.0062	4.534E-08

rsID	EFAL	NEFAL	EAF	B	SE	P-value
rs10790162	A	G	0.09103	0.2305	0.0065	1E-200
rs1260326	T	C	0.4129	0.1148	0.0034	1E-200
rs12678919	A	G	0.8786	0.1702	0.0056	1.82E-199
rs2954022	C	A	0.5303	0.078	0.0033	2.23E-113
rs11974409	A	G	0.8061	0.0899	0.0042	1.36E-100
rs4587594	G	A	0.69	0.0694	0.0035	3.503E-82
rs676210	G	A	0.7691	0.0733	0.0039	3.284E-71
rs10401969	T	C	0.92876	0.121	0.0065	9.702E-70
rs439401	C	T	0.6201	0.0659	0.0038	1.423E-66
rs174535	C	T	0.3628	0.047	0.0034	1.733E-41
rs11820504	C	T	0.2032	0.0604	0.0044	1.095E-39
rs4810479	C	T	0.2876	0.0474	0.0038	2.067E-34
rs1321257	G	A	0.4063	0.0402	0.0034	5.986E-31
rs588136	C	T	0.2058	0.0495	0.0041	3.365E-30
rs247616	C	T	0.7071	0.0393	0.0037	1.123E-25
rs2247056	C	T	0.7823	0.0378	0.0039	3.861E-21
rs2043085	T	C	0.3681	0.0327	0.0034	7.809E-20
rs442177	T	G	0.5528	0.0309	0.0033	1.316E-18
rs10761762	T	C	0.533	0.027	0.0033	1.061E-17
rs16948098	A	G	0.0409	0.08	0.0089	4.838E-17
rs9686661	T	C	0.1768	0.0379	0.0044	2.541E-16
rs6882076	C	T	0.6662	0.0286	0.0035	1.513E-15
rs13389219	C	T	0.591	0.0271	0.0034	2.598E-15
rs2972146	T	G	0.6227	0.0281	0.0034	2.974E-15
rs998584	A	C	0.5145	0.0293	0.0037	3.424E-15
rs634869	T	C	0.438	0.0272	0.0033	1.776E-14
rs11613352	C	T	0.8087	0.028	0.0039	9.397E-14
rs6995541	G	A	0.3219	0.0265	0.0037	1.343E-12
rs6831256	G	A	0.409	0.0258	0.0035	1.602E-12
rs1832007	A	G	0.8681	0.0327	0.0047	1.718E-12
rs645040	T	G	0.7691	0.0293	0.004	1.83E-12
rs11057408	G	T	0.6372	0.0258	0.0035	2.049E-12
rs12676857	C	T	0.1544	0.0332	0.0046	7.291E-12
rs3761445	A	G	0.6148	0.0232	0.0034	8.062E-12
rs2068888	G	A	0.5092	0.0241	0.0034	1.682E-11
rs10440120	C	A	0.8325	0.0306	0.0044	5.343E-11
rs4719841	G	A	0.3826	0.0232	0.0034	8.864E-11
rs2250802	A	G	0.6807	0.023	0.0037	1.21E-10
rs2239520	G	A	0.6266	0.0236	0.0037	4.144E-10
rs7248104	G	A	0.5831	0.0222	0.0034	5.045E-10
rs749671	G	A	0.6055	0.0211	0.0034	6.106E-10
rs2665357	C	A	0.5092	0.0212	0.0033	8.327E-10
rs12748152	T	C	0.07124	0.0372	0.0059	1.1E-09
rs731839	G	A	0.3417	0.0224	0.0036	2.651E-09
rs3760627	C	T	0.4683	0.0189	0.0034	5.293E-09
rs287621	T	C	0.2704	0.0222	0.0037	7.671E-09
rs4738684	A	G	0.3522	0.0205	0.0035	8.819E-09
rs8077889	C	A	0.2441	0.0252	0.0042	9.879E-09
rs10501321	T	C	0.686	0.0216	0.0035	1.412E-08
rs17513135	T	C	0.2322	0.022	0.0039	1.633E-08
rs38855	A	G	0.5264	0.0187	0.0033	2.109E-08
rs3198697	C	T	0.6174	0.0198	0.0034	2.207E-08
rs719726	T	C	0.529	0.0199	0.0035	2.486E-08
rs6029143	C	T	0.94195	0.0388	0.0071	4.933E-08

rsID	EFAL	NEFAL	EAF	B	SE	P-value
rs7412	C	T	0.93404	0.3736	0.0096	1E-200
rs6511720	G	T	0.90237	0.1851	0.0059	1E-200
rs646776	T	C	0.7876	0.1272	0.0042	4.77E-187
rs515135	C	T	0.7823	0.1238	0.0046	6.38E-151
rs11591147	G	T	0.98285	0.3341	0.0173	8.827E-86
rs6544713	T	C	0.2942	0.0773	0.004	1.685E-81
rs12916	C	T	0.4314	0.0684	0.0036	4.547E-74
rs2954029	A	T	0.5317	0.0622	0.0035	2.421E-65
rs2228603	C	T	0.92876	0.1217	0.0069	1.049E-62
rs964184	G	C	NA	0.1214	0.0076	2.838E-55
rs1883025	C	T	0.7573	0.0671	0.0042	5.749E-53
rs10468017	T	C	0.2757	0.0617	0.004	7.232E-48
rs780093	T	C	0.4129	0.0515	0.0036	2.591E-42
rs579459	C	T	0.215	0.062	0.0044	8.828E-42
rs6882076	C	T	0.6662	0.0508	0.0037	5.354E-41
rs2000999	A	G	0.1847	0.0617	0.0044	6.804E-41
rs1535	A	G	0.6372	0.0497	0.0037	8.624E-39
rs9306897	T	C	0.3034	0.0488	0.0037	7.515E-37
rs9987289	G	A	0.9248	0.0842	0.0063	1.844E-36
rs247616	T	C	0.2929	0.0499	0.004	4.47E-32
rs2156552	T	A	0.8219	0.057	0.0047	1.247E-31
rs7534572	G	C	0.69	0.0629	0.0055	3.597E-28
rs558971	G	A	0.5303	0.0398	0.0036	7.025E-28
rs2235367	G	A	0.4565	0.0357	0.0035	7.219E-25
rs1800961	C	T	0.9657	0.1062	0.0101	1.342E-24
rs4738684	A	G	0.3522	0.0392	0.0037	1.116E-23
rs11753995	A	G	0.1464	0.0489	0.0048	1.839E-23
rs75687619	T	G	0.02375	0.1592	0.0153	3.609E-22
rs9391858	G	A	0.1939	0.0495	0.005	7.204E-22
rs7551981	T	G	0.595	0.0358	0.0037	7.497E-22
rs2073547	G	A	0.1939	0.0456	0.0047	3.83E-21
rs2642438	G	A	0.7454	0.037	0.004	1.283E-18
rs2244608	G	A	0.3391	0.0313	0.0037	9.618E-18
rs6016373	A	G	0.6266	0.0319	0.0036	1.002E-17
rs3184504	C	T	0.5343	0.0318	0.0037	1.623E-17
rs7616006	A	G	0.5554	0.0315	0.0036	8.406E-17
rs12670798	C	T	0.2243	0.0364	0.0041	9.478E-17
rs2737252	G	A	0.7441	0.0331	0.0039	1.634E-16
rs2255141	A	G	0.3193	0.0314	0.0039	6.514E-16
rs3757354	C	T	0.7902	0.0348	0.0042	2.215E-15
rs2814982	C	T	0.8931	0.0441	0.0057	3.678E-15
rs11220462	A	G	0.1425	0.0474	0.0058	5.487E-15
rs8103315	A	C	0.1359	0.0422	0.0055	5.942E-15
rs6603981	T	C	0.8061	0.0351	0.0043	7.846E-15
rs633695	G	A	0.285	0.0433	0.0058	1.054E-14
rs11153594	C	T	0.6082	0.029	0.0036	1.266E-14
rs10832962	T	C	0.719	0.0315	0.0039	1.539E-14
rs11802413	T	C	0.5369	0.0287	0.0035	1.576E-14
rs4988235	G	A	0.4763	0.0308	0.004	3.975E-14
rs581080	C	G	0.8206	0.0377	0.0047	1.022E-13
rs7832643	T	G	0.405	0.0289	0.0037	3.121E-13
rs1800562	G	A	0.95383	0.0565	0.0077	1.905E-12
rs2287623	G	A	0.405	0.0273	0.0036	4.085E-12
rs6504872	T	C	0.4723	0.025	0.0035	6.987E-12

rs2315065	A	C	0.08707	0.1102	0.0158	1.098E-11
rs11789603	T	C	0.08971	0.0427	0.0062	1.439E-11
rs2738459	A	C	0.5554	0.0387	0.0057	2.109E-11
rs10904908	G	A	0.4538	0.025	0.0036	2.601E-11
rs6573778	T	C	0.471	0.0263	0.0039	2.958E-11
rs2277862	C	T	0.8681	0.0349	0.0052	5.256E-11
rs6818397	T	G	0.4129	0.0254	0.0039	9.51E-11
rs1997243	G	A	0.1306	0.0332	0.005	2.719E-10
rs314253	T	C	0.6649	0.0233	0.0037	2.808E-10
rs10088180	A	G	0.3219	0.0228	0.004	6.018E-10
rs3780181	A	G	0.94723	0.0442	0.0071	6.668E-10
rs12412743	C	T	0.847	0.0298	0.0047	6.976E-10
rs181360	T	G	0.8008	0.0278	0.0043	7.319E-10
rs6709904	A	G	0.8865	0.0545	0.0083	8.395E-10
rs11563251	T	C	0.1253	0.0368	0.0059	1.266E-09
rs4752805	G	A	0.2467	0.0251	0.0041	1.617E-09
rs4530754	A	G	0.5818	0.0228	0.0035	1.678E-09
rs4883201	A	G	0.8865	0.035	0.0056	1.742E-09
rs11694172	G	A	0.2164	0.0277	0.0041	1.951E-09
rs9376090	T	C	0.7282	0.0254	0.004	2.595E-09
rs10773003	A	G	0.08839	0.0369	0.0058	4.083E-09
rs17526895	A	G	0.92216	0.042	0.0067	5.777E-09
rs10900221	A	G	0.2731	0.0255	0.0041	7.963E-09
rs4253772	T	C	0.1187	0.0322	0.0058	9.852E-09
rs2066714	C	T	0.1201	0.0442	0.0076	1.136E-08
rs112201728	T	C	0.05805	0.0581	0.0099	1.195E-08
rs7640978	C	T	0.8945	0.0376	0.0066	1.658E-08
rs9272775	C	T	0.2823	0.0317	0.0055	2.125E-08
rs13315871	G	A	0.91953	0.0355	0.0061	3.48E-08
rs2030746	T	C	0.3984	0.0199	0.0037	3.603E-08
rs2886232	T	C	0.1201	0.0358	0.0062	3.874E-08
rs281393	C	T	0.6266	0.0322	0.0055	4.256E-08
rs386003	T	G	0.1992	0.0344	0.0058	4.519E-08
rs138777	A	G	0.3483	0.0214	0.0037	4.74E-08

Supplementay Table 10: Genetic variants associated with fasting glucose

rsID	EFAL	NEFAL	EAF	B	SE	P-value
rs10830963	C	G	0.3	-0.078	0.0025	1E-200
rs560887	C	T	0.326	0.071	0.0025	1.4E-178
rs6975024	T	C	0.2	-0.061	0.0029	2.88E-99
rs2191349	G	T	0.467	-0.029	0.0021	1.28E-42
rs1260326	C	T	0.42	0.029	0.0021	2.17E-41
rs11558471	A	G	0.252	0.029	0.0023	7.8E-37
rs17168486	C	T	0.173	-0.031	0.0028	3.17E-28
rs4502156	T	C	0.42	0.022	0.0021	1.38E-25
rs11607883	G	A	0.469	0.021	0.0021	6.32E-24
rs7903146	C	T	0.279	-0.022	0.0024	2.71E-20
rs174576	C	A	0.345	0.02	0.0022	1.18E-18
rs11708067	A	G	0.226	0.023	0.0026	1.3E-18
rs11195502	C	T	0.075	0.032	0.0037	1.97E-18
rs10811661	T	C	0.199	0.024	0.0028	5.65E-18
rs1280	T	C	0.137	0.026	0.0031	8.56E-18
rs4869272	C	T	0.323	-0.018	0.0022	1.02E-15
rs11619319	A	G	0.212	-0.02	0.0024	1.33E-15
rs749067	C	T	0.376	-0.017	0.0022	6.12E-15
rs983309	G	T	0.097	-0.026	0.0033	6.29E-15
rs10814916	C	A	0.434	0.016	0.0022	2.26E-13
rs6943153	C	T	0.279	-0.015	0.0022	1.63E-12
rs882020	C	T	0.121	-0.021	0.003	3.04E-12
rs17712208	T	A	0.041	-0.051	0.0074	3.22E-12
rs479661	A	G	0.142	-0.019	0.0028	8.56E-12
rs11603334	G	A	0.129	0.019	0.0028	1.12E-11
rs6113722	G	A	0.04	0.035	0.0053	2.49E-11
rs16913693	T	G	0.017	0.043	0.0066	3.51E-11
rs3829109	G	A	0.345	0.017	0.0027	1.13E-10
rs3783347	G	T	0.219	0.017	0.0026	1.32E-10
rs2302593	C	G	0.475	0.014	0.0023	9.26E-10
rs9368222	C	A	0.281	-0.014	0.0023	0.000000001
rs10747083	A	G	0.25	0.013	0.0023	7.57E-09
rs6072275	G	A	0.142	-0.016	0.0028	1.66E-08
rs7651090	A	G	0.296	-0.013	0.0023	1.75E-08
rs11715915	C	T	0.274	0.012	0.0022	0.000000049

rsID	EFAL	NEFAL	EAF	B	SE	P-value
rs1121980	G	A	0.482	-0.02	0.0025	8.36E-16
rs983309	G	T	0.097	-0.029	0.0038	3.81E-14
rs1260326	C	T	0.42	0.019	0.0026	3.84E-14
rs9884482	T	C	0.35	-0.016	0.0024	1.4E-11
rs7903146	C	T	0.279	0.018	0.0028	6.13E-11
rs10195252	C	T	0.442	-0.016	0.0026	4.87E-10
rs1167800	A	G	0.451	0.016	0.0026	2.61E-09
rs2820436	C	A	0.322	0.015	0.0026	4.36E-09
rs2745353	T	C	0.45	0.014	0.0025	5.48E-09
rs860598	A	G	0.136	0.018	0.0032	1.64E-08
rs731839	A	G	0.341	-0.014	0.0026	1.72E-08
rs4865796	A	G	0.292	0.015	0.0026	2.09E-08
rs2972143	G	A	0.371	0.014	0.0026	3.15E-08
rs1530559	A	G	0.403	0.014	0.0026	3.37E-08

Supplementay Table 12: Genetic variants associated with body mass index

rsID	EFAL	NEFAL	EAF	B	SE	P-value
rs1421085	C	T	0.45	0.0803	0.003	2.17E-158
rs6567160	C	T	0.2833	0.0562	0.0035	6.684E-59
rs13021737	A	G	0.125	-0.0604	0.0039	5.439E-54
rs13130484	C	T	0.5667	-0.0398	0.003	8.011E-41
rs543874	G	A	0.2667	0.0497	0.0037	2.287E-40
rs943005	T	C	0.1	0.0444	0.0038	4.524E-31
rs11030104	A	G	0.8	0.0416	0.0037	6.658E-30
rs7531118	T	C	0.3917	-0.0331	0.003	1.88E-28
rs7138803	G	A	0.5583	-0.032	0.003	5.115E-26
rs10182181	A	G	0.5	-0.0309	0.0029	8.071E-26
rs3888190	A	C	0.3583	0.0311	0.003	3.454E-25
rs1516725	T	C	0.0917	-0.0448	0.0044	1.394E-24
rs11672660	C	T	0.825	0.0339	0.0038	7.911E-19
rs13329567	T	C	0.2167	-0.0307	0.0035	1.526E-18
rs3817334	C	T	0.55	-0.0256	0.003	1.168E-17
rs2112347	G	T	0.375	-0.0254	0.003	1.96E-17
rs7144011	T	G	0.275	0.0274	0.0035	6.045E-15
rs13078960	T	G	0.8167	-0.029	0.0038	1.423E-14
rs2183825	C	T	0.2917	0.0241	0.0032	2.223E-14
rs7550711	T	C	0.0339	0.0659	0.0087	5.059E-14
rs11165643	C	T	0.425	-0.0221	0.003	1.434E-13
rs17066856	C	T	0.1333	-0.0371	0.005	2.003E-13
rs657452	A	G	0.4167	0.0227	0.0031	2.123E-13
rs12429545	G	A	0.9	-0.0324	0.0044	3.152E-13
rs12286929	G	A	0.4333	0.0211	0.0029	5.443E-13
rs13107325	C	T	0.8833	-0.0472	0.0066	1.064E-12
rs7903146	T	C	0.25	-0.0235	0.0033	1.103E-12
rs1016287	T	C	0.325	0.0228	0.0033	4.355E-12
rs10840100	G	A	0.725	0.0206	0.003	6.666E-12
rs2060604	T	C	0.5583	0.0203	0.003	9.46E-12
rs10132280	A	C	0.3333	-0.0221	0.0033	1.401E-11
rs17094222	C	T	0.2083	0.0249	0.0037	2.186E-11
rs12448257	G	A	0.775	-0.0246	0.0037	3.898E-11
rs17381664	C	T	0.425	0.0201	0.0031	4.568E-11
rs7599312	G	A	0.7083	0.0214	0.0033	4.73E-11
rs9926784	T	C	0.7917	0.0249	0.0038	8.548E-11
rs2365389	C	T	0.6583	0.0195	0.003	1.346E-10
rs9579083	G	C	0.7667	-0.0295	0.0046	1.426E-10
rs16851483	G	T	0.9083	-0.0478	0.0075	1.85E-10
rs1167827	A	G	0.4583	-0.02	0.0031	1.975E-10
rs10733682	A	G	0.425	0.0188	0.003	2.455E-10
rs6457796	T	C	0.7417	-0.0209	0.0033	2.535E-10
rs12940622	A	G	0.4583	-0.0183	0.0029	3.636E-10
rs879620	C	T	0.4083	-0.0244	0.0039	3.939E-10
rs1928295	C	T	0.425	-0.0182	0.0029	4.318E-10
rs2820292	A	C	0.4917	-0.0181	0.0029	5.452E-10
rs4889606	G	A	0.3583	-0.0187	0.003	6.58E-10
rs6804842	A	G	0.425	-0.0183	0.003	8.016E-10
rs13191362	A	G	0.8	0.0285	0.0047	1.092E-09
rs9540493	G	A	0.55	-0.0182	0.0031	3.952E-09
rs1528435	T	C	0.5833	0.0175	0.003	4.774E-09
rs17001654	C	G	0.8417	-0.0304	0.0052	5.031E-09
rs11727676	C	T	0.075	-0.0365	0.0063	6.247E-09
rs4740619	T	C	0.5333	0.017	0.0029	6.356E-09

rs9374842	T	C	0.7417	0.0196	0.0034	7.198E-09
rs17724992	A	G	0.6917	0.0196	0.0034	7.787E-09
rs7715256	G	T	0.45	0.0168	0.0029	8.851E-09
rs12986742	C	T	0.5	0.0207	0.0036	8.924E-09
rs11057405	A	G	0.0917	-0.0304	0.0053	1.22E-08
rs2890652	T	C	0.875	-0.0279	0.0049	1.242E-08
rs7899106	A	G	0.95	-0.0379	0.0067	1.269E-08
rs2033529	G	A	0.2583	0.0183	0.0032	1.449E-08
rs9304665	A	T	0.7	0.0243	0.0043	1.594E-08
rs2836754	C	T	0.65	0.0169	0.003	1.605E-08
rs891389	C	T	0.675	-0.0209	0.0037	1.617E-08
rs6477694	C	T	0.3583	0.0169	0.003	1.705E-08
rs1000940	G	A	0.225	0.0184	0.0033	1.812E-08
rs14810	C	G	0.325	-0.0183	0.0033	1.923E-08
rs3849570	A	C	0.3667	0.0183	0.0033	1.933E-08
rs6713510	A	G	0.4833	0.0164	0.0029	1.974E-08
rs6091540	C	T	0.725	0.0185	0.0033	2.138E-08
rs977747	T	G	0.4667	0.0168	0.003	2.182E-08
rs1441264	A	G	0.55	0.0172	0.0031	2.959E-08
rs17203016	G	A	0.2	0.0211	0.0038	3.406E-08
rs2176598	T	C	0.2	0.0185	0.0033	3.469E-08
rs13201877	A	G	0.9167	-0.0236	0.0043	4.285E-08
rs3736485	A	G	0.425	0.016	0.0029	4.524E-08
rs3800229	T	G	0.6917	0.0175	0.0032	4.95E-08
rs1460676	T	C	0.7833	-0.0209	0.0038	4.978E-08

Supplementay Table 13: Genetic variants associated with Waist Hip Ratio adjusted Body mass index

rsID	EFAL	NEFAL	EAF	B	SE	P-value
rs1421085	C	T	0.45	0.043	0.0033	4.3E-38
rs9491696	G	C	0.525	0.037	0.0032	1.4E-30
rs998584	A	C	0.475	0.029	0.0036	4.8E-15
rs1294410	C	T	0.625	0.025	0.0033	2E-14
rs1106529	G	A	0.275	-0.028	0.0038	2.2E-13
rs13424740	T	C	0.5333	0.024	0.0032	2.6E-13
rs459193	A	G	0.2167	0.027	0.0037	3.4E-13
rs10783615	G	A	0.1333	0.034	0.0046	3.7E-13
rs1563355	T	C	0.3136	-0.031	0.0044	1.7E-12
rs11663816	C	T	0.3167	0.026	0.0037	3.1E-12
rs3786897	G	A	0.4083	0.022	0.0033	2.5E-11
rs10842708	G	A	0.1833	0.024	0.0036	3.1E-11
rs10245353	A	C	0.1833	0.027	0.0041	3.5E-11
rs2371767	G	C	0.7917	0.024	0.0037	1.2E-10
rs1128249	G	T	0.5583	0.02	0.0033	7.8E-10
rs12549058	G	T	0.0583	0.037	0.006	8.3E-10
rs17451107	T	C	0.625	0.021	0.0034	1.2E-09
rs1440372	C	T	0.7417	0.022	0.0036	2.7E-09
rs7973683	C	A	0.6167	0.02	0.0034	3.3E-09
rs4823006	A	G	0.5333	0.019	0.0033	3.3E-09
rs714515	G	A	0.4583	0.019	0.0033	5.9E-09
rs6736025	G	T	0.5917	0.024	0.0042	7.2E-09
rs16996700	T	C	0.7	0.021	0.0036	8.8E-09
rs2972164	C	T	0.5	0.019	0.0033	0.00000001
rs2287019	C	T	0.85	0.025	0.0044	0.000000011
rs6743060	C	A	0.125	-0.025	0.0043	0.000000015
rs13130484	C	T	0.5667	-0.019	0.0033	0.000000015
rs4929927	G	A	0.725	0.019	0.0033	0.000000016
rs4640244	G	A	0.375	0.02	0.0037	0.00000003
rs879048	A	C	0.8	0.022	0.004	0.000000036
rs2398893	A	G	0.6833	0.02	0.0036	0.00000004

rsID	EFAL	NEFAL	EAF	B	SE	P-value
rs247617	A	C	0.285998	0.197243	0.011688	7.9E-63
rs261291	C	T	0.374904	0.144314	0.010905	2.58E-39
rs6507939	C	A	0.838753	0.108424	0.014268	5.03E-14
rs11632618	A	G	0.061853	0.174133	0.024085	7.76E-13
rs174594	A	C	0.58921	0.071714	0.010504	1.32E-11
rs73424577	G	A	0.035619	0.186041	0.030161	9.78E-10
rs1883025	T	C	0.194349	-0.079839	0.013321	2.86E-09
rs144064722	G	A	0.025304	0.203723	0.035039	8.3E-09
rs75835816	C	G	0.019619	-0.22095	0.038801	1.67E-08
rs1461729	G	A	0.861933	0.086363	0.015193	1.77E-08
rs4860951	T	A	0.351356	0.073728	0.013224	3.29E-08

Supplementay Table 15: Genetic variants associated with apolipoprotein B

rsID	EFAL	NEFAL	EAF	B	SE	P-value
rs7412	T	C	0.055855	-0.427553	0.025979	4.39E-59
rs11591147	T	G	0.031101	-0.437941	0.035298	2.5E-34
rs142130958	A	G	0.105527	-0.199648	0.016743	7.78E-32
rs964184	C	G	0.861008	-0.165767	0.014266	2.58E-30
rs1367117	A	G	0.287972	0.108852	0.011191	9.99E-22
rs10056811	A	G	0.341411	0.085783	0.010571	1.35E-15
rs190934192	A	G	0.023998	-0.319724	0.040849	1.3E-14
rs3005923	A	G	0.027652	-0.282948	0.03695	4.73E-14
rs629301	T	G	0.779917	0.090052	0.012193	3.56E-13
rs2980875	G	A	0.483896	-0.069675	0.009993	6.68E-12
rs1260326	C	T	0.638553	-0.066785	0.010393	2.51E-10
rs150617279	A	T	0.110384	-0.112384	0.017688	3.97E-10
rs4722043	C	G	0.417089	-0.065942	0.010669	1.17E-09
rs6756629	A	G	0.078334	-0.113253	0.018566	1.9E-09
rs635634	T	C	0.198293	0.073971	0.012612	7.71E-09
rs115849089	A	G	0.106662	-0.099572	0.017126	1.04E-08
rs1883711	C	G	0.056795	0.144092	0.02526	1.95E-08
rs144064722	G	A	0.025304	0.199001	0.035059	2.29E-08
rs1081105	C	A	0.020011	0.222868	0.039281	2.32E-08
rs182695896	C	A	0.019349	0.236546	0.041781	2.49E-08
rs2495477	G	A	0.418981	-0.061897	0.011013	3.14E-08

Supplementay Table 16: Genetic variants associated with cigarettes per day

rsID	EFAL	NEFAL	EAF	B	SE	P-value
rs12914385	T	C	0.3965	1.0235	0.0828	4.229E-35
